# *Trametes robiniophila* Murr Sensitizes Gastric Cancer Cells to 5-Fluorouracil by Modulating Tumor Microenvironment

**DOI:** 10.3389/fphar.2022.911663

**Published:** 2022-05-17

**Authors:** Jing-Li Xu, Li Yuan, Can Hu, Chun-Yan Weng, Han-Dong Xu, Yun-Fu Shi, Ling Huang, Jie-Er Ying, Zhi-Yuan Xu, Jiang-Jiang Qin, Xiang-Dong Cheng

**Affiliations:** ^1^ The First Clinical Medical College of Zhejiang Chinese Medical University, Hangzhou, China; ^2^ The Cancer Hospital of the University of Chinese Academy of Sciences (Zhejiang Cancer Hospital), Institute of Basic Medicine and Cancer (IBMC), Chinese Academy of Sciences, Hangzhou, China; ^3^ Zhejiang Provincial Research Center for Upper Gastrointestinal Tract Cancer, Zhejiang Cancer Hospital, Hangzhou, China; ^4^ Key Laboratory of Prevention, Diagnosis and Therapy of Upper Gastrointestinal Cancer of Zhejiang Province, Hangzhou, China

**Keywords:** gastric cancer, *Trametes robiniophila* Murr, 5-FU, MDSCs, CD8^+^ T cells, NK cells

## Abstract

*Trametes robiniophila Murr* (TRM) is a traditional Chinese medicine which has been used in clinics for enhancing immunity and improving the efficacy of chemotherapy. However, the mechanisms of action of TRM are unknown. In the previous study, we found that the *Trametes robiniophila Murr* n-butanol extract (TRMBE) comprises the major bioactive components of TRM. In the present study, we aimed to assess the combinational effects of TRMBE and 5-fluorouracil (5-FU) on the treatment of gastric cancer (GC) and explore its mechanism of action. It was found that TRMBE significantly potentiated the anticancer activity of 5-FU and prolonged the survival time of mice bearing Mouse Forestomach Carcinoma (MFC) xenograft tumors. We observed that the combination of TRMBE and 5-FU decreased the risk of liver metastasis *in vivo*. Furthermore, the combination of TRMBE and 5-FU reduced the levels of immune cytokines IL-6, IL-10, and TGF-β and increased the level of IFN-γ in peripheral blood. This combination therapy also significantly decreased the levels of polymorphonuclear myeloid-derived suppressor cells (PMN-MDSCs) and PD-1-positive CD8^+^ T cells and increased the levels of NK cells in tumor microenvironment (TME). However, TRMBE treatment was unable to enhance the chemosensitivity of GC to 5-FU *in vivo* after the depletion of CD8^+^ T and NK cells. Taken together, our results demonstrate that TRMBE can reshape the TME of GC by regulating PMN-MDSCs, CD8^+^ T cells, and NK cells, therefore improving the therapeutic effects of 5-FU. This study suggests that the combination of TRMBE and 5-FU could enhance immunity and could be a promising approach for GC treatment.

## Introduction

Gastric cancer (GC) is the fifth most common malignancy (Sung et al., 2021). Despite the decline in incidence due to the improvement in living standard and medical advances, GC ranks as the third cause of cancer-related death and remains a serious threat globally (Bray et al., 2018). Surgery combined with chemotherapy is the most common strategy for the management of GC because more than 60% of patients have already developed local or distant metastasis at diagnosis (Yuan et al., 2020). Fluorouracil, paclitaxel, and platinum drugs are the most commonly used chemotherapeutic drugs (Wang FH. et al., 2019). However, poor or even no response to chemotherapy and severe adverse effects frequently occur in most GC patients, resulting in treatment failure (Xu et al., 2020; Zhang et al., 2020). Thus, new therapeutic strategies such as combination therapies are needed.

Immune cells infiltrated into the tumor microenvironment (TME) play indispensable roles in GC cell initiation, growth, and metastasis (Kaymak et al., 2021). CD8^+^ cytotoxic T cells (CTLs) and natural killer (NK) cells can kill tumor cells by secreting large amounts of perforin, protease granzyme B, and interferon-γ (IFN-γ) (Cózar et al., 2021). However, some immunosuppressive cytokines, such as interleukin-6 (IL-6), IL-10, and transforming growth factor (TGF)-β, promote the recruitment and maturation of immunosuppressive cells (Bunt et al., 2007). Myeloid-derived suppressor cells (MDSCs) are one of the major types of these immunosuppressive cells. In humans and mouse, MDSCs include polymorphonuclear MDSCs (PMN-MDSCs) and monocytic MDSCs (M-MDSCs) subpopulations. Normally, murine PMN-MDSCs are labelled as CD11b^+^ Ly6G^+^ Ly6C^low^ or CD11b^+^ Gr-1^high^ and M-MDSCs are labelled as CD11b^+^ Ly6G^−^ Ly6C^+^ or CD11b^+^ Gr-1^low^; while human MDSCs are labelled as HLA-DR^−^ CD11b^+^ CD33^+^ CD15^+^ and HLA-DR^−^ CD11b^+^ CD33^+^ CD14^+^ (Youn and Gabrilovich, 2010; Filipazzi et al., 2012). MDSCs can significantly inhibit the activity of CTLs and NK cells (Hart et al., 2011; Yang et al., 2020a). Besides, MDSCs express a high level of programmed cell death-Ligand 1 (PD-L1) and increase the expression of programmed cell death 1 (PD-1) on the cell membrane surface of CD8^+^ T cells, leading to the anergy of CTLs (Liu et al., 2018; Groth et al., 2019).

Increasing evidence has demonstrated that traditional Chinese medicine (TCM) can enhance immunity, prevent tumor recurrence and metastasis and prolong survival of patients (Yang et al., 2020b; Lu et al., 2020; Wang et al., 2021). Multiple studies have found that TCM alone or in combination with conventional chemotherapy or radiotherapy can reduce tumor size and alleviate tumor-related symptoms, thus improving the quality of life of patients with tumors (He et al., 2021; Markowitsch et al., 2021; Wang et al., 2021). *Trametes robiniophila Μurr* (TRM), also known as Huaier, is a TCM which has an approximate 1,600-year history of use (Wang Y. et al., 2019). Clinical studies have shown that TRM can prolong the recurrence-free survival of patients with hepatic carcinoma and breast cancer (Chen et al., 2018; Liu XY. et al., 2020). It has been reported that TRM may exert anti-tumor activity through regulating immune cells, including CD4^+^ T cells (Li et al., 2015), NK cells (Li et al., 2015), macrophages (Li et al., 2016), and dendritic cells (DCs) (Pan et al., 2020). We have recently found that the *Trametes robiniophila Murr* n-butanol extract (TRMBE) comprises the major bioactive components of TRM and exhibits potent anticancer activity in gastric cancer (GC) and adenocarcinoma of the esophagogastric junction (Wang Y. et al., 2019; Yuan et al., 2021). However, the effect of combinational use of TRMBE and other chemotherapeutic drugs is unclear.

In the present study, we investigate whether TRMBE can enhance the therapeutic effect of chemotherapeutic drugs on GC by regulating immunity. We combined TRMBE with 5-fluorouracil (5-FU) for the treatment of GC. The results showed that TRMBE significantly enhances the anticancer activity of 5-FU, inhibits the development of GC and reduces the risk of liver metastasis. Furthermore, TRMBE combined with 5-FU could reduce immune cytokines IL-6, IL-10 and TGF- β, and increase IFN- γ in the peripheral blood. The combination of TRMBE and 5-FU significantly decreased the number of PMN-MDSCs and the expression of PD-1 on CD8^+^ T cells, and increased the number of NK cells in the TME. Finally, we demonstrated that CD8^+^ T cells and NK cells are the cells that were responsible for TRMBE to enhance the anticancer effect of 5-FU. These results provided evidence for the potential application of TRMBE in combination with 5-FU for GC therapy.

## Materials and Methods

### Cell Line and Mice

Mouse Forestomach Carcinoma (MFC) cell line was purchased from the Chinese Academy of Sciences (Beijing, China). Cells were cultured in RPMI-1640 medium supplemented with 10% FBS, 100 U/ml penicillin and 100 µg/ml streptomycin at 37°C with 5% CO_2_. Cells were subcultured every 2 days. Male 615 mice (4–6 weeks old, 18–25 g weight) were obtained from the Tianjin Institute of Hematology and housed in a specific pathogen-free facility of Zhejiang Chinese Medical University Laboratory Animal Research Centre. All animal experiments were performed following the guidelines approved by the Institutional Animal Care and Use Committee of Zhejiang Chinese Medical University.

### Animal Models

To establish the subcutaneous GC model, 5×10^5^ MFC cells were injected subcutaneously into the left axilla of recipient mice. Tumor dimensions were measured with a Vernier caliper every day, and tumor volumes were calculated according to formula 1/2a×b^2^, where a and b represented the larger and smaller dimensions of the tumor, respectively. The tumor-bearing mice were randomized into vehicle and different treatment groups. Among them, part of the mice in each group (*n* = 6 mice per group) were used to evaluate the therapeutic effect and another part in each group (*n* = 10 mice per group) were used for survival analysis. The survival time was calculated from the first day after injection of cells to the day when the mice showed one of the following situations and had to be sacrificed. The mice were euthanized separately if they 1) tumor volume exceeding 1000 mm^3^, 2) weight loss exceeding 20% compared to the baseline, 3) exhibited severely impaired activity or treatment-related severe adverse events that caused pain or distress and that could not be ameliorated with analgesics. The remaining half of the mice were sacrificed together when the tumor volume of any mouse reached 1500 mm^3^.

To establish the liver metastasis model, the spleen was accessed through a 1-cm incision in the upper left lateral abdomen of anesthetized mice. 5×10^5^ MFC cells were suspended in 100 µl PBS and injected into the spleen parenchyma. To avoid intrasplenic tumor growth, the spleen was removed 10 min later (Jiao et al., 2021). 2 weeks later, mice were euthanized and their livers were removed. Liver weights and the numbers of metastatic nodules were counted.

For evaluating the depletion of CD8^+^ T cell or NK cells, mice bearing MFC tumors were injected intraperitoneally with 100 µg neutralizing anti-CD8a (BioXcell, cat: BE0117) or 100 µg neutralizing anti-NK1.1 antibody (BioXcell, cat: BE0036) biweekly for 2 weeks. The control groups received an equivalent amount of saline.

### Treatment of Model Mice With TRMBE and 5-FU

TRM was purchased from Bozhou Medical Materials Co., Ltd. (Anhui, China). And TRMBE was obtained as described previously (Wang Y. et al., 2019; Yuan et al., 2021). Specifically, the fruiting bodies of TRM were powdered and reflux-extracted with heated 90% ethanol twice (2 h each time). The extract solution was filtered and combined, evaporated, concentrated, and vacuum drying into ethanol extract powder. Subsequently, ethanol extract powder was extracted with petroleum ether, ethyl acetate, n-butanol, 90% ethanol, and distilled water sequentially. Five different powders of TRM extract were obtained by concentrating and recovering the solvent on a rotary evaporator, freezing, and vacuum drying. According to the information analysis of LC-MS for TRMBE (Supplementary Figures S1–S8), 21 potential compounds were identified (Supplementary Table S1) and used as quality control standards. Finally, the obtained n-butanol extract powder was stored in a −18°C refrigerator. The powder was dissolved in saline: DMSO (95: 5, v/v) and sterilized with a 0.22-μm filter to obtain a 40 mg/ml stock solution. 5-FU was obtained from Good Laboratory Practice bioscience (cat NO. GC14466) and dissolved in saline with ultrasonic vibration and warming to obtain 1 mg/ml solution. Fresh dilutions were made for each experiment. The concentration of TRMBE is according to previously study (Yuan et al., 2021). All animal model experiments were divided mice into four groups as follows: 1) Vehicle group: normal saline containing 5% DMSO (200 µl, gavage, once a day) and normal saline (200 µl, intraperitoneal (i.p.) injection, once a day); 2) TRMBE group: TRMBE solution (100 mg/kg, gavage, once a day) and normal saline (200 µl, i.p. injection, once a day); 3) 5-FU group: normal saline containing 5 %DMSO (200 µl, gavage, once a day) and 5-FU solution (10 mg/kg, i.p. injection, once a day); 4) Combine group: TRMBE solution (100 mg/kg, gavage, once a day) and 5-FU solution (10 mg/kg, i.p. injection, once a day).

### Spectral Flow Cytometry

To analyze the immune cell components, spleens and tumors of MFC tumor-bearing mice were sampled. Spleen lymphocytes were prepared as described by Li et al. (2020). Briefly, fresh spleen was gently scraped with sterile cover glasses in PBS and the cells were filtered with a 70 µm strainer after erythrocytes were lysed. Tumor tissue was dissociated into single-cell suspensions according to the protocol of the Tumor Dissociation Kit (Miltenyi Biotec, cat: 130-096-730). Single cells were stained with fluorescein-conjugated monoclonal antibodies for 30 min at 4°C, and subsequently analyzed on Flow Cytometer (Cytek Aurora 3,000). The monoclonal antibodies include CD45 (Biolegend, cat: 103138), CD3 (Biolegend, cat: 100210), CD4 (Thermo, cat: 48-0041-82), CD8 (BD Pharmingen, cat: 557959), CD25 (Biolegend, cat: 102008), PD-1 (Biolegend, cat: 109118), NK1.1 (Biolegend, cat: 108710), CD11b (Biolegend, cat: 101228), Gr-1 (Biolegend, cat: 108426), CD19 (Biolegend, cat: 115543), CD45R/B220 (Biolegend, cat: 103244), CD11c (Biolegend, cat: 117348), F4/80 (Biolegend, cat: 123118), and MHC-Ⅱ (Biolegend, cat: 107643).

### Enzyme-Linked Immunosorbent Assay (ELISA)

Peripheral blood of mice was collected and placed at room temperature for 30 min, and then centrifuged at 5000 RPM for 10 min. The supernatant was taken for ELISA analysis. The serum levels of cytokines (including IL-1b, IL-2, IL-4, IL-5, IL-6, IL-10, IL-12p70, IL-13, IL-17, IL-17F, IL-21, IL-22, IL-23, IL-28, IFN-γ, MIP-3a, TGF-β, and TNF-α) were measured by commercial ELISA kits (RayBiotech, cat: QAM-TH17-1-2) according to the manufacturer’s protocols. The light absorbance values were read at the recommended wavelengths using microplate reader (Infinite^®^ M1000 Pro).

### Statistical Analysis

Statistical analyses were performed using The SPSS version 23.0 software and GraphPad Prism 6.0 software. Comparisons across groups were performed by ANOVA or *t*-test, and Kaplan-Meier survival curves were compared by log-rank test. Flow cytometry data were analyzed by FlowJo V.10. A *p* < 0.05 was considered to be statistically significant. ^∗^ *p* value <0.05, ^∗∗^ *p* value <0.01 and ^∗∗∗^ *p* value <0.001.

## Results

### TRMBE Sensitizes the Therapeutic Effect of 5-FU on MFC Tumor

**FIGURE 1:**
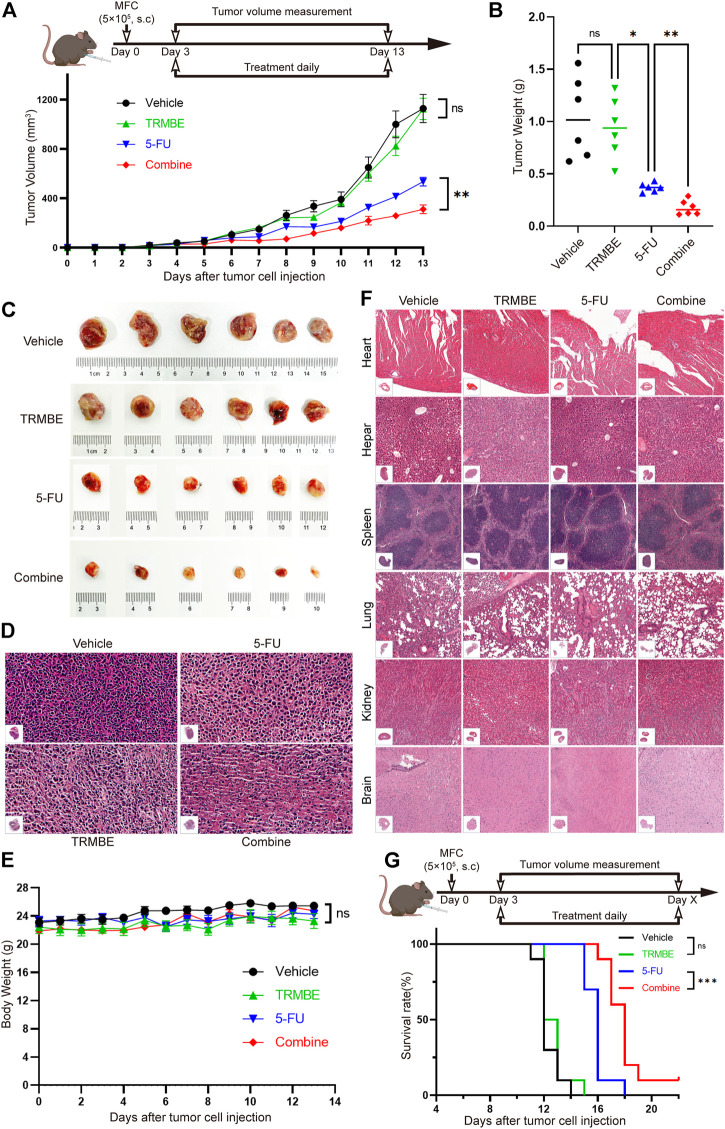
The combination of TRMBE and 5-FU suppressed gastric cancer growth and prolonged overall survival rate of tumor-bearing mice. **(A)** The treatment schedule and tumor volume of the subcutaneous gastric cancer mouse model. A total of 5×10^5^ MFC cells were separately injected into the right flanks of 615 mice. Mice were randomly divided into vehicle group, TRMBE group, 5-FU group, and combined group (*n* = 6 in every group). Tumor growths were measured. **(B,C)** Tumor weights in different groups and images of subcutaneous MFC tumor. **(D)** H&E staining of subcutaneous MFC tumor tissues. **(E)** Average body weights of mice bearing MFC tumor. **(F)** H&E staining of major representative organs. **(G)** The survival curves. Overall survival of mice was shown (*n* = 10 in every group). Data are presented as means ± SEM. NS, *p* > 0.05; *p < 0.05; ***p < 0.001.

To determine the anti-tumor effect of TRMBE *in vivo*, the MFC tumor-bearing mice were randomized in groups and treated with different therapeutic strategies. As shown in Figures 1A–C, TRMBE showed a slight inhibitory effect on MFC tumor with no statistical significance (*p* > 0.05). The 5-FU and the combined therapy exhibited significant inhibition on MFC tumor in terms of tumor size and weight, and the tumor inhibitory rate of the combined group was significantly higher than that of the 5-FU group. Furthermore, H&E staining revealed the therapy efficiency (Figure 1D): tumor cells in the vehicle group and TRMBE group showed irregular morphology, high nucleus/cytoplasm ratio, heterochromatic and polymorphism; In the tumors of 5-FU group and especially the combined group, tumor cells exhibited greater nuclear pyknosis and cytoplasmic lysis. Importantly, the combined treatment did not affect the body weights of the mice compared with other three treatments (Figure 1E), and H&E staining on major organs showed no abnormality in different groups (Figure 1F), suggesting that TRMBE alone and combined therapy did not cause host toxicity in the treatment period. Meanwhile, we performed a survival experiment and found that the combination of 5-FU and TRMBE significantly prolonged the survival time of the tumor-bearing mice (Figure 1G, combine, 18 ± 1.63 days; vehicle, 12.3 ± 0.82 days; 5-FU, 15.9 ± 0.88 days; TRMBE, 12.7 ± 0.95 days). These results indicated that TRMBE can enhance the antitumor effect of 5-FU and the combined therapy can prolong the survival time of the tumor-bearing mice.

### The Combination of TRMBE and 5-FU Inhibits MFC Tumor Liver Metastasis

**FIGURE 2:**
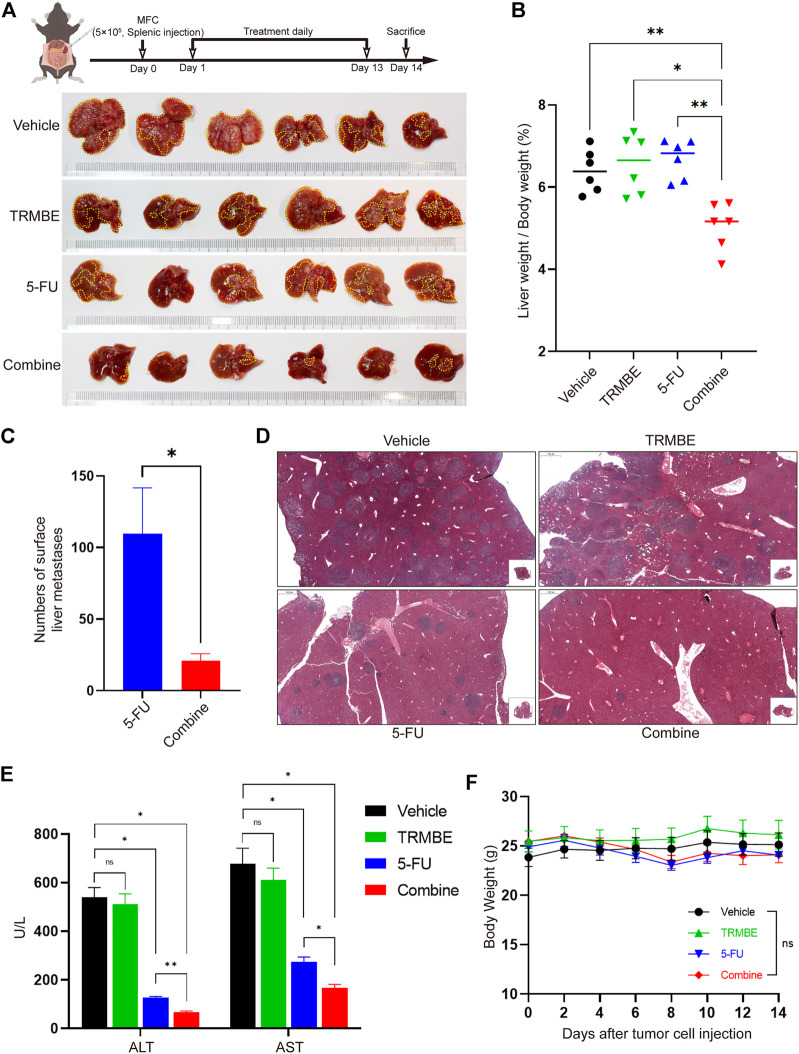
The combination of TRMBE and 5-FU inhibited liver metastases of gastric cancer. **(A)** The treatment schedule of liver metastases model and the representative images of liver metastatic tumors in different groups (*n* = 6 in every group). **(B)** The proportion of liver weight to body weight in four groups. **(C)** The number of liver metastatic tumor nodules in 5-FU and combined group. **(D)** H&E staining of tumor metastasis in the livers. **(E)** The levels of ALT and AST in these four groups. **(F)** The body weight of mice in these four groups. Data are analyzed by ANOVA or *t*-test and presented as means ± SEM. NS, *p* > 0.05; *p < 0.05; **p < 0.01.

To explore whether the combination of TRMBE and 5-FU could reduce the risk of liver metastasis, we established a MFC liver-metastasis mouse model and treated the mice with different therapeutic regimens from the second day postoperatively. At the end of the intervention, it was found that most of the livers in the vehicle and TRMBE treatment groups are occupied by MFC metastatic tumor nodules, while the number of liver metastatic tumor nodule in the mice treated with 5-FU or combined therapy was significantly reduced (Figure 2A). In addition, the combined treatment group showed a lower liver weight/body weight proportion compared with the other groups (Figure 2B). Notably, the combined treatment significantly reduced the number of tumor metastatic nodules compared with the 5-FU treatment (Figures 2A,C,D). In addition, the levels of aspartate aminotransferase (ALT) and alanine aminotransferase (AST) in vehicle and TRMBE group were significantly higher than normal, indicating that the liver function of mice were severely impaired due to metastatic MFC tumor (Figure 2E). As expected, the 5-FU therapy decreased the levels of ALT and AST, and the combined therapy exhibited a greater ability to rescue the liver function than 5-FU therapy without affecting the body weight of mice (Figures 2E,F). These findings suggested that the TRMBE can sensitize the effect of 5-FU and inhibited liver metastasis of GC.

### The Combination of TRMBE and 5-FU Enhances Immunity by Regulating Immune Cytokines

**FIGURE 3:**
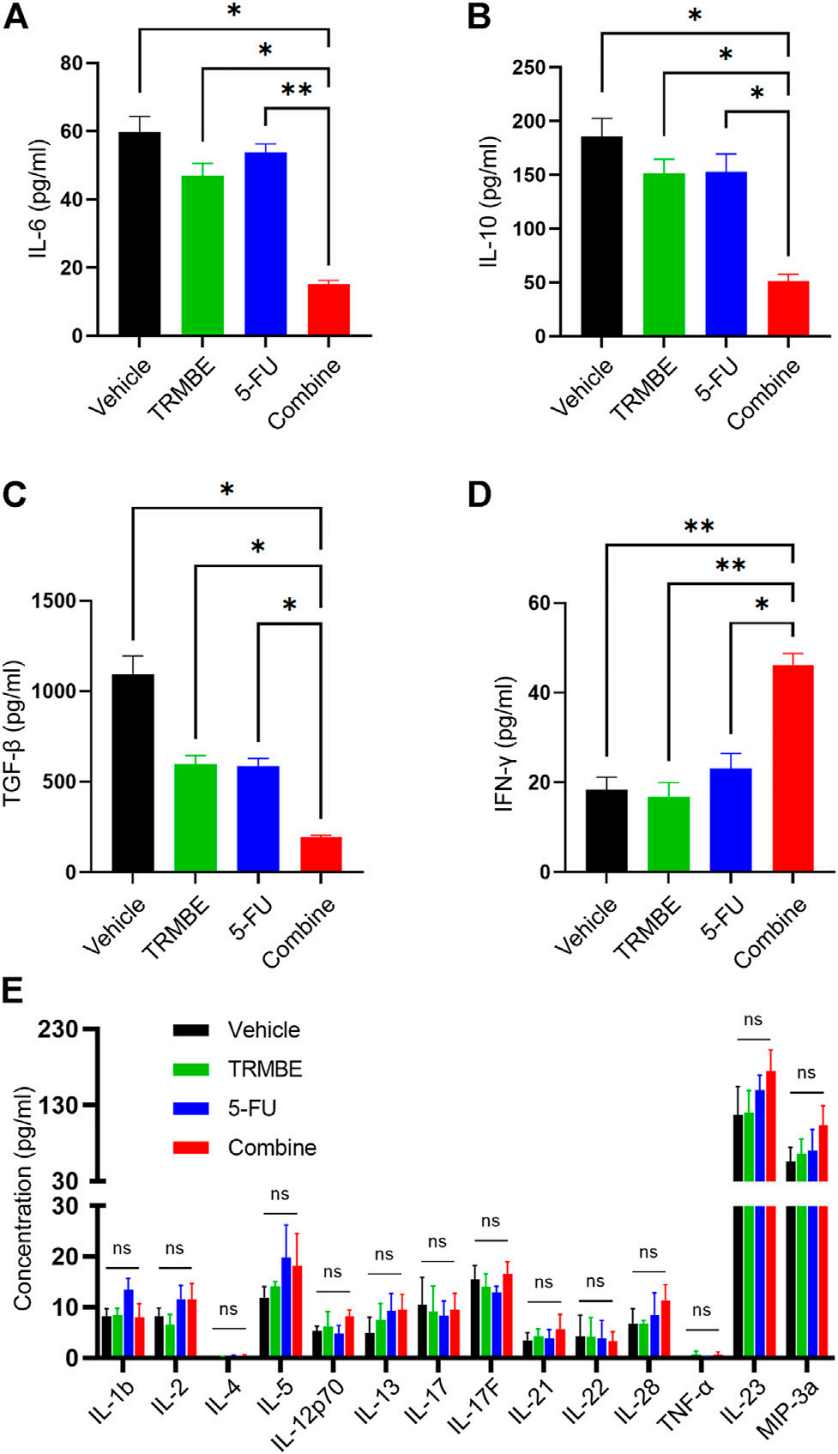
The combination of TRMBE and 5-FU regulates the levels of cytokines in peripheral blood, including IL-6 **(A)**, IL-10 **(B)**, TGF-β **(C)**, IFN-γ **(D)** and other cytokines **(E)**. Data are analyzed by ANOVA and presented as means ± SEM. NS, *p* > 0.05; *p < 0.05; **p < 0.01.

Previous studies have demonstrated that multiple immunosuppressive cytokines, such as IL-6, IL-10 and TGF-β promote the maturation and recruitment of immunosuppressive cells (Bunt et al., 2007) and impair the anti-tumor functions of NK cells and CD8^+^ T cells (Fleming et al., 2018). To investigate the regulatory effect of TRMBE in combination with 5-FU in the immune system, we used ELISA kit to detect the levels of cytokines in peripheral blood. As presented in Figures 3A–C, the combined therapy significantly reduced the levels of IL-6, IL-10, and TGF-β in serum compared with the other groups, while neither 5-FU nor TRMBE monotherapy group significantly reduced these levels compared with the vehicle group. It was known that IFN-γ exerts an indispensable role in tumor killing and immunoregulation of NK cells and TCLs (St Paul and Ohashi, 2020; Liu et al., 2021). As expected, the combination of TRMBE and 5-FU significantly increased the IFN-γ level in serum, while treatment with TRMBE or 5-FU alone did not affect the serum level of IFN-γ (Figure 3D). As for other cytokines, there was no statistically significant difference among the four groups (Figure 3E). These results suggested that the combined therapy can reduce multiple immunosuppressive cytokines in peripheral circulation, including IL-6, IL-10 and TGF-β, and promote the secretion of IFN-γ to promote the immunity of the mice.

### The Combined Treatment Decreases the Number of PMN-MDSCs and PD-1+ CD8^+^ T Cells, and Increases the Number of NK Cells in the TME

**FIGURE 4:**
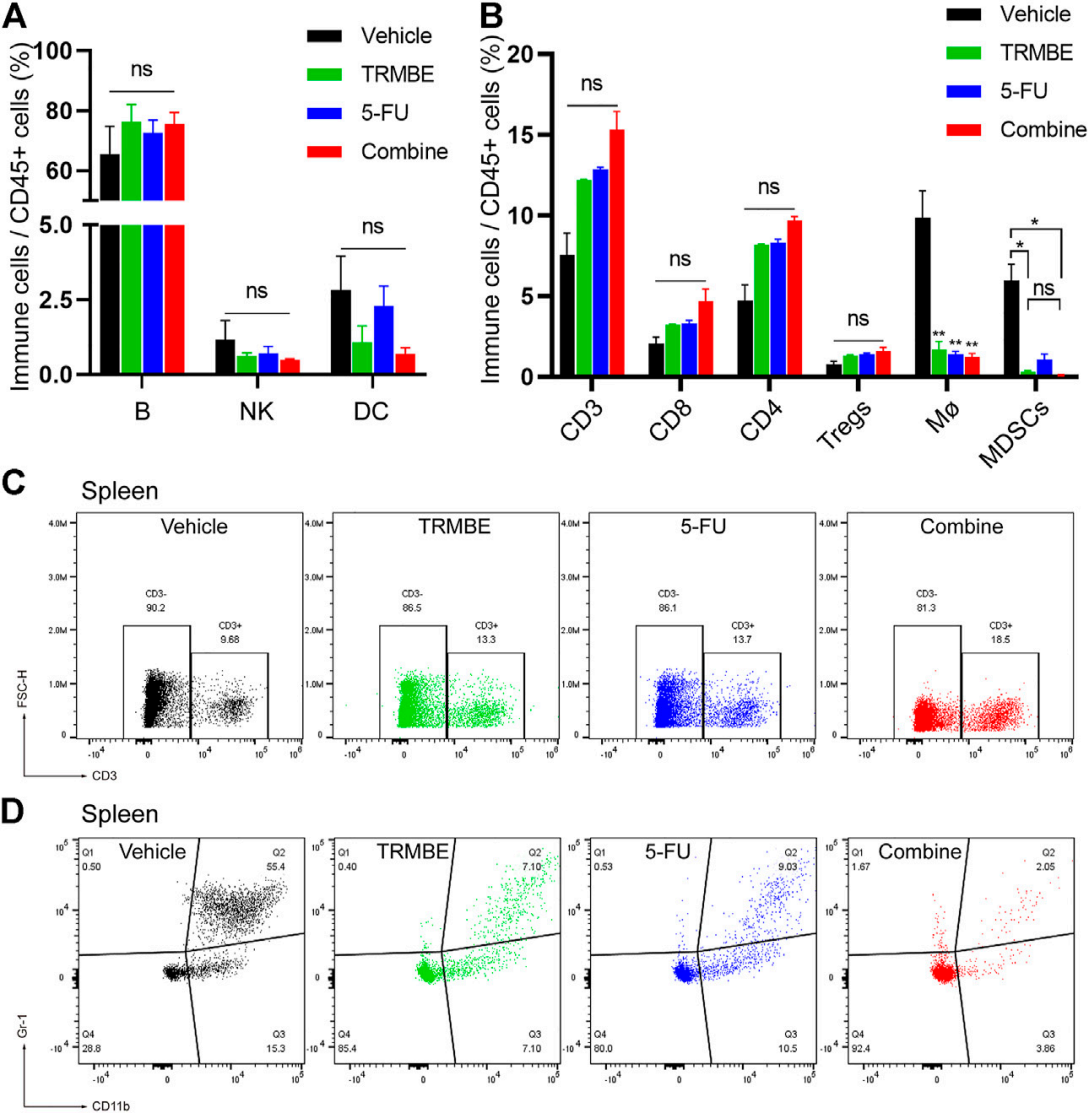
The proportion of immune cells in the spleen of subcutaneous tumor mice. **(A,B)** Spectral Flow Cytometry showing the proportion of tumor-infiltrating immune cells in the spleens of mice after treatment. **(C,D)** Representative flow cytometry gating images showing the percentages of T cells and MDSCs. Data are presented as means ± SEM. NS, *p* > 0.05; *p < 0.05; **p < 0.01.

To investigate the immune regulation of TRMBE and 5-FU on the MFC tumor, immune cells in spleen were examined by Spectral Flow Cytometry. Figure 4A demonstrated that the percentage of splenic B lymphocytes, NK cells, and DC cells were not changed in TRMBE group, 5-FU group, or combined group (*p* > 0.05). Similarly, the numbers of CD3^+^ T cells, CD4^+^ T cells, and CD8^+^ T cells were not significantly changed (Figures 4B,C). However, compared with the vehicle group, the number of macrophages were significantly reduced in the other three groups (*p* < 0.01) (Figure 4B). Moreover, TRMBE alone, 5-FU alone, and the combined treatment induced a significant reduction of PMN-MDSCs compared with vehicle groups (*p* < 0.05), whereas the combined treatment group did not strengthen this effect than the groups treated with TRMBE alone and 5-FU alone (*p* > 0.05) (Figures 4B,D). These results indicated that TRMBE, 5-FU or the combination of both did not affect the immune cells in the spleen.

**FIGURE 5:**
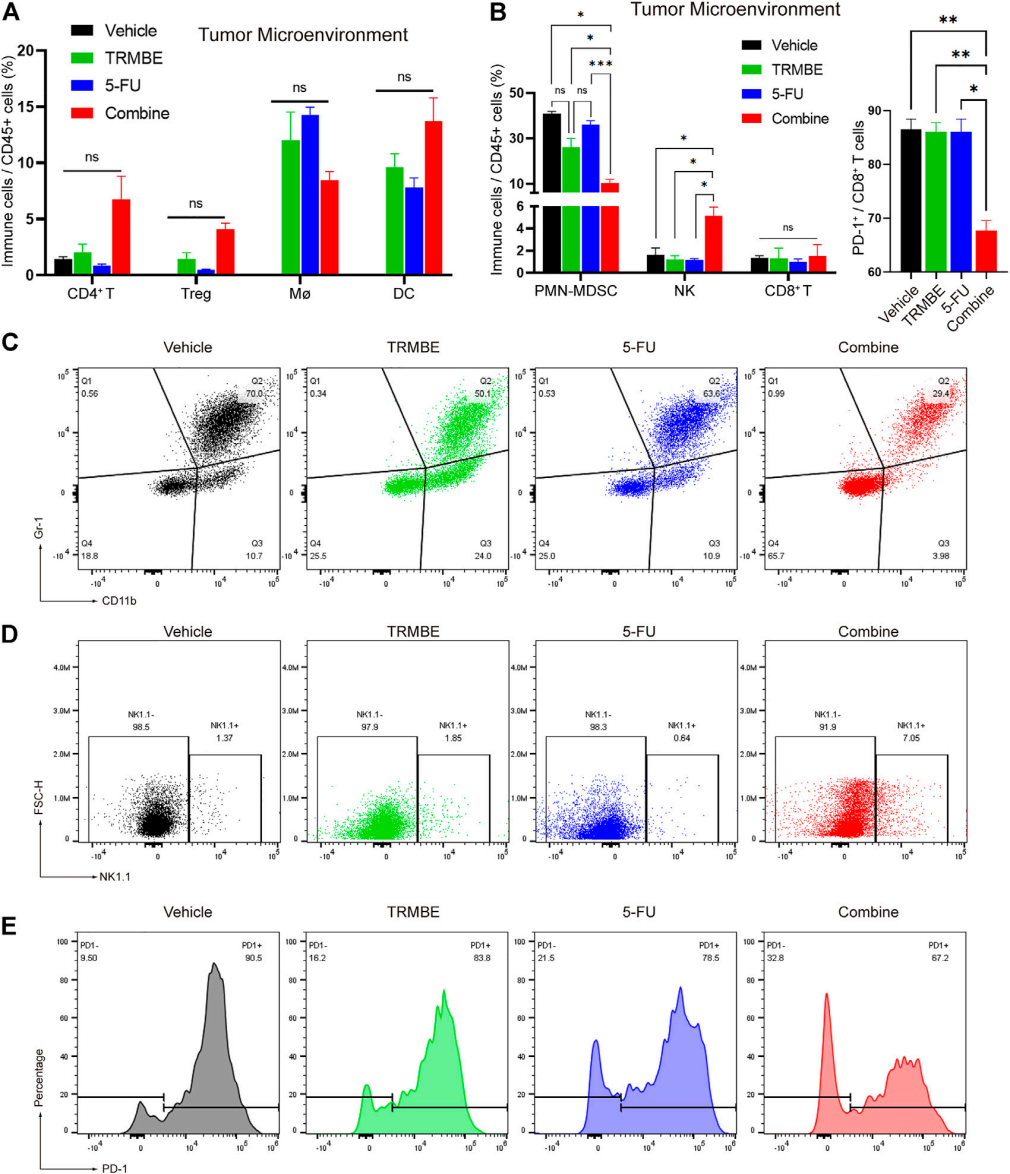
The combination of TRMBE and 5-FU regulates the proportion of tumor-infiltrating immune cells in mice. **(A,B)** Spectral Flow Cytometry showing the proportion of tumor-infiltrating immune cells in the tumor tissues of mice after treatment. **(C,D)** Representative flow cytometry gating images showing the percentages of MDSCs and NK cells. **(E)** The expression of PD-1 on CD8^+^ T cells after treatment detected by Spectral Flow Cytometry. Data are analyzed by ANOVA and presented as means ± SEM. NS, *p* > 0.05; *p < 0.05; **p < 0.01; ***p < 0.001.

To further evaluate the immunomodulatory effect induced by TRMBE and 5-FU, we next examined the immune cells infiltrating into the MFC tumors by Spectral Flow Cytometry. The Spectral Flow Cytometry results showed that macrophages, DC cells, CD4^+^ T cells and regulatory cells (Tregs) had no significant change among the four groups (Figure 5A). Notably, compared with the other groups, the combination of TRMBE and 5-FU reduced the percentage of PMN-MDSCs (*p* < 0.01) (Figures 5B,C). In addition, the combined therapy induced a significant increase in the proportion of NK cells, while neither 5-FU alone or TRMBE alone had this effect (Figures 5B,D). Although there was no significant difference in the percentage of CD8^+^ cytotoxic T cells among the four treatment groups, the expression of PD-1 on CD8^+^ T cells in the combined treatment group was significantly lower than that in the other groups (Figures 5B,E). In other words, only the combined therapy can induce the percentage of NK cells and reduce the expression of PD-1 on CD8^+^ T cells to activate CD8^+^ cytotoxic T cells, while TRMBE or 5-FU alone didn’t have this effect. These results suggested that TRMBE combined with 5-FU can significantly reduce the percentage of PMN-MDSCs and the expression of PD-1 on CD8^+^ T cells, and increase the levels of NK cells, reshaping TME.

### CD8^+^ T Cells and NK Cells are the Effector Cells of TRMBE in Enhancing the Anticancer Effect of 5-FU

**FIGURE 6:**
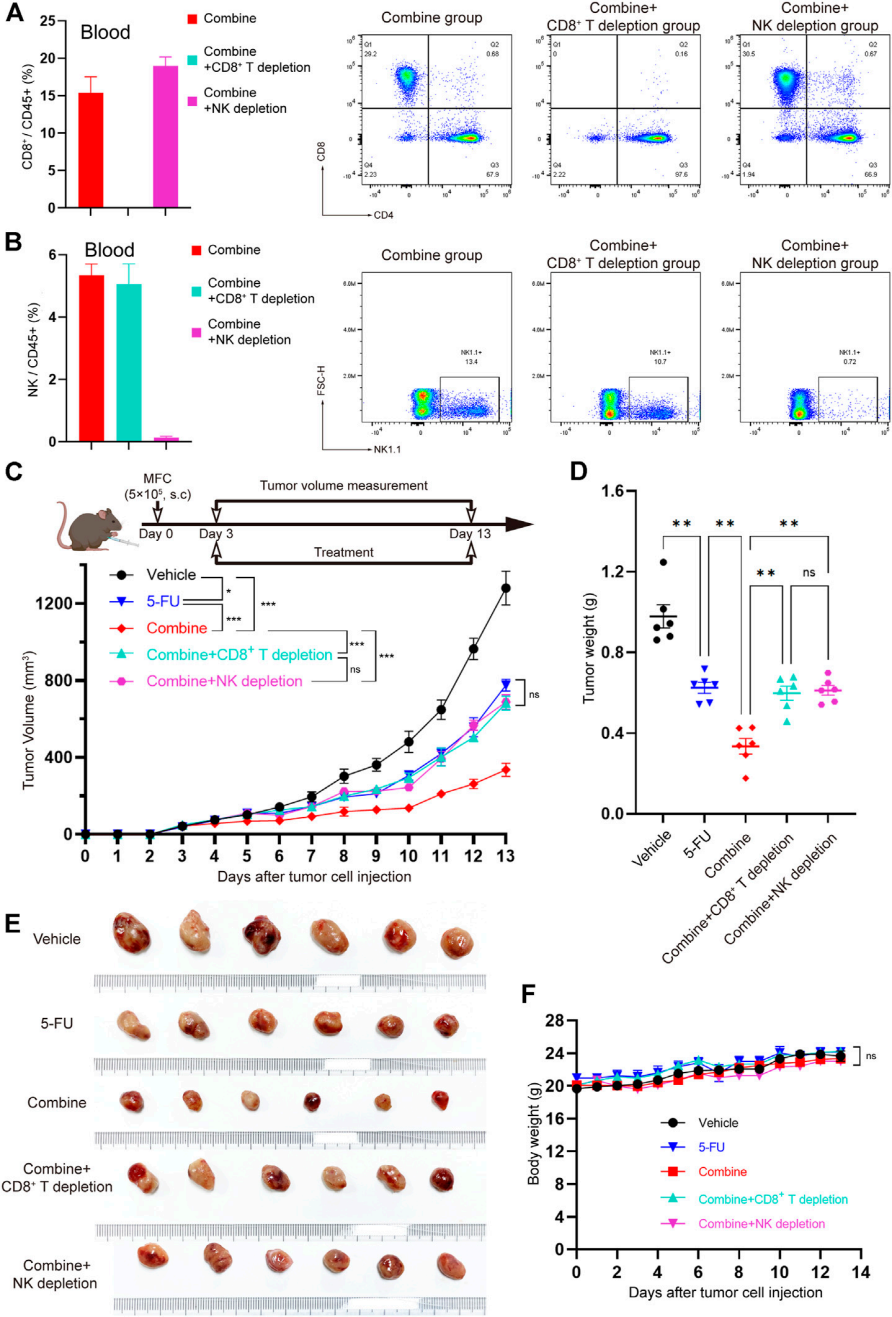
The CD8^+^ T cells and NK cells were the effector cells of TRMBE in enhancing the anticancer effect of 5-FU. **(A,B)** Percentage of CD8^+^ T cells and NK cells in blood at day 13 in the mice (left panel) and representative flow cytometry of CD8^+^T cells and NK cells at day 13 in the mice (right panel). **(C)** The treatment schedule of subcutaneous gastric cancer model and tumor volume. Mice were randomly divided into vehicle group, 5-FU group, combined group, combined + CD8^+^ T depletion group, and combined + NK depletion group. Tumor growths were measured (*n* = 6 in every group). **(D,E)** Tumor weights in different groups and representative images of subcutaneous MFC tumor. **(F)** Average body weight of mice bearing MFC tumor. Data are analyzed by ANOVA and presented as means ± SEM. NS, *p* > 0.05; *p < 0.05; **p < 0.01; ***p < 0.001.

To further verify whether CD8^+^ T cells and/or NK cells are effector killer cells that are responsible for inhibiting the development of tumors by TRMBE treatment, we used the CD8a-neutralizing antibody or NK1.1-neutralizing antibody to block CD8^+^ T cells or NK cells, respectively, in the mice bearing MFC tumors. We randomly divided the tumor-bearing mice into five groups and treated them with different strategies. We first assessed the CD8^+^ T cell and NK cell depletion efficiency of CD8a-neutralizng and NK1.1-neutralizing antibodies. Spectral Flow Cytometry results showed that CD8^+^ T cells and NK cells in murine blood were successfully and completely depleted by anti-CD8 antibody and anti-NK antibody (Figures 6A,B). As expected, depletion of CD8^+^ T cell abolished the enhanced anti-tumor effect of TRMBE and 5-FU combined treatment, and depletion of NK cell showed the same results (Figures 6C–F). Notably, with the depletion of CD8^+^ T cell and NK cell, the tumor volumes and weights of combined therapy group return to the levels of the 5-FU therapy group. These results suggested that TRMBE enhances the antitumor effect of 5-FU by inducing the infiltration of CD8^+^ T cells and NK cells, promoting the killing effect of them.

## Discussion

Tumor invasion and metastasis are key prognostic factors of GC patients. The peritoneum and liver are the most common sites of GC metastasis (Renzulli et al., 2020). There are some first-line chemotherapeutic combination regimens for the treatment of advanced GC, including Trastuzumab-based regimes for HER2-positive cases, fluoropyrimidine-based regimens, and platinum-based regimens (Wang FH. et al., 2019; Blair et al., 2020). However, the treatment outcome using these regimes remains poor (Bray et al., 2018; Fitzmaurice et al., 2019). The PD-1 monoclonal antibodies have been approved in current clinical trials for advanced GC based on the results of previous phase 2 and 3 trials (Kang et al., 2017; Fuchs et al., 2018). Although some studies have shown that PD-1 or PD-L1 monoclonal antibodies show good therapeutic prospects for some patients with advanced gastric cancer, most of the ineffective treatments limit their clinical application (Shitara et al., 2018; Song et al., 2020). Therefore, comprehensive therapeutic strategies consisting of chemotherapy, targeted therapy, immunotherapy, and TCM treatment are urgently needed.

In the TME, antigen-presenting cells (APCs) present cancer neoantigens to naive CD8^+^ T cells and secrete cytokine interleukin-12 (IL-12) to active CD8^+^ T cells. Primed CD8^+^ T cells subsequently recognize the neoantigens presented by tumor cells through the major histocompatibility complex (MHC) or human leukocyte antigen (HLA) class I molecules, resulting in elimination of tumor cells (Croft et al., 1994; Wculek et al., 2020; Jhunjhunwala et al., 2021). In addition, interferon-γ (IFN-γ) secreted from CTLs can upregulate MHC-I and reprogram the suppressive cells to modulate adaptive immune responses in TME (Zaidi and Merlino, 2011; Overacre-Delgoffe et al., 2017). Natural killer (NK) cells exhibit similar cytotoxicity to kill targets that do not express sufficiently large numbers of MHC-I (Cózar et al., 2021). With the loss of the main ligands for NK-cell inhibitory receptors and the activation of natural cytotoxicity receptors (NCR), NK cells can secret perforin, protease granzyme B, and IFN-γ to kill tumor cells (Cózar et al., 2021).

Under the action of tumor-derived factors, some myeloid cells might be reprogrammed and fail to mature. These immature cells, known as MDSCs, may infiltrate into tumor microenvironment and impair the normal physiological activities of CTLs and NK cells, contributing to tumor immune escape (Krishnamoorthy et al., 2021). Specifically, several cytokines and growth factors released by immune cells and tumor cells, such as IL-1β (Elkabets et al., 2010), IL-6 (Bunt et al., 2007), and TGF-β (Fleming et al., 2018) promote the development of MDSCs. MDSCs express Fas-ligand (FasL) and produce Arg-1, iNOS, IDO, and NOX2 to induce apoptosis (Zhu et al., 2017) and cell cycle arrest (Morello et al., 2016) of T cells. Among them, IDO degrades L-tryptophan into kynurenine, inducing PD-1 expression in CD8^+^ T cells through AhR activation (Liu et al., 2018). Meanwhile, MDSCs express high levels of PD-L1, leading to anergy of CTLs (Groth et al., 2019). Furthermore, MDSCs inhibit the proliferation, cytokine secretion, and granzyme-B production of NK cells in a contact-dependent manner or by high levels of ROS and iNOS (Hart et al., 2011; Yang et al., 2020a). In the TME, high levels of TGF-β and IL-10 produced by MDSCs can significantly impair the function of CTLs and NK cells (Hart et al., 2011; Yang et al., 2020a). Thus, targeting MDSCs may restore the immunity of CTLs and NK cells. Increasing evidence has shown that targeting MDSCs can significantly improve the efficacy of anti-cancer therapy (Dominguez et al., 2017; Yu et al., 2019; Groth et al., 2021), which provides a new strategy for comprehensive cancer therapy.

TCM has been used frequently in clinics as tumor adjuvant therapy to improve the treatment effect and reduce the side effects of chemotherapy (Xiang et al., 2019). Previously studies have demonstrated that several naturally bioactive compounds isolated from herbal medicines can modulate immune checkpoints in tumors (Liu Y. et al., 2020; Yin et al., 2020). Besides, multiple natural products modulate the TME by regulating the activities of several types of immune cells, including T lymphocytes, B lymphocytes, Tregs, DCs, and NK cells, thereby exert anti-tumor function (Sellam et al., 2020; Jiang et al., 2021; Zhong et al., 2022). However, in clinical treatment, the use of natural products is limited due to individual differences in patients, tumor heterogeneity, TME differences and other characteristics. Because their mechanisms are not fully explained, there is still a great lag in developing natural products as tumor immunotherapy. *Trametes robiniophila Murr* (TRM), a promising TCM, has shown beneficial effect in the multi-center, randomized, controlled, phase IV trial and has been recommended as postoperative adjuvant therapy in patients with liver cancer by the Chinese society of clinical oncology guidelines. The cancer inhibitory effect of TRM on various cancer types has been documented in recent years (Xu et al., 2017; Ma et al., 2018; Fang et al., 2019). According to the analyses of HPLC (high-performance liquid chromatography) and SDS-PAGE (polyacrylamide gel electrophoresis), the most effective ingredients of TRM are identified as proteoglycans, which include 41.53% polysaccharides (key ingredient), 12.93% amino acids, and 8.72% water (Wang W. et al., 2019; Hu et al., 2019). We used different solvents (etroleum ether, ethylacetate, n-butanol, ethanol, and water phases) to extract TRM, and yielded five types of extracts. Our previous studies have demonstrated that the TRMBE has the most potent anticancer activity (Wang Y. et al., 2019; Yuan et al., 2021). Then, we applied LC-MS to analyze TRMBE and found several potential compounds (Supplementary Figures S1–S8).

**FIGURE 7:**
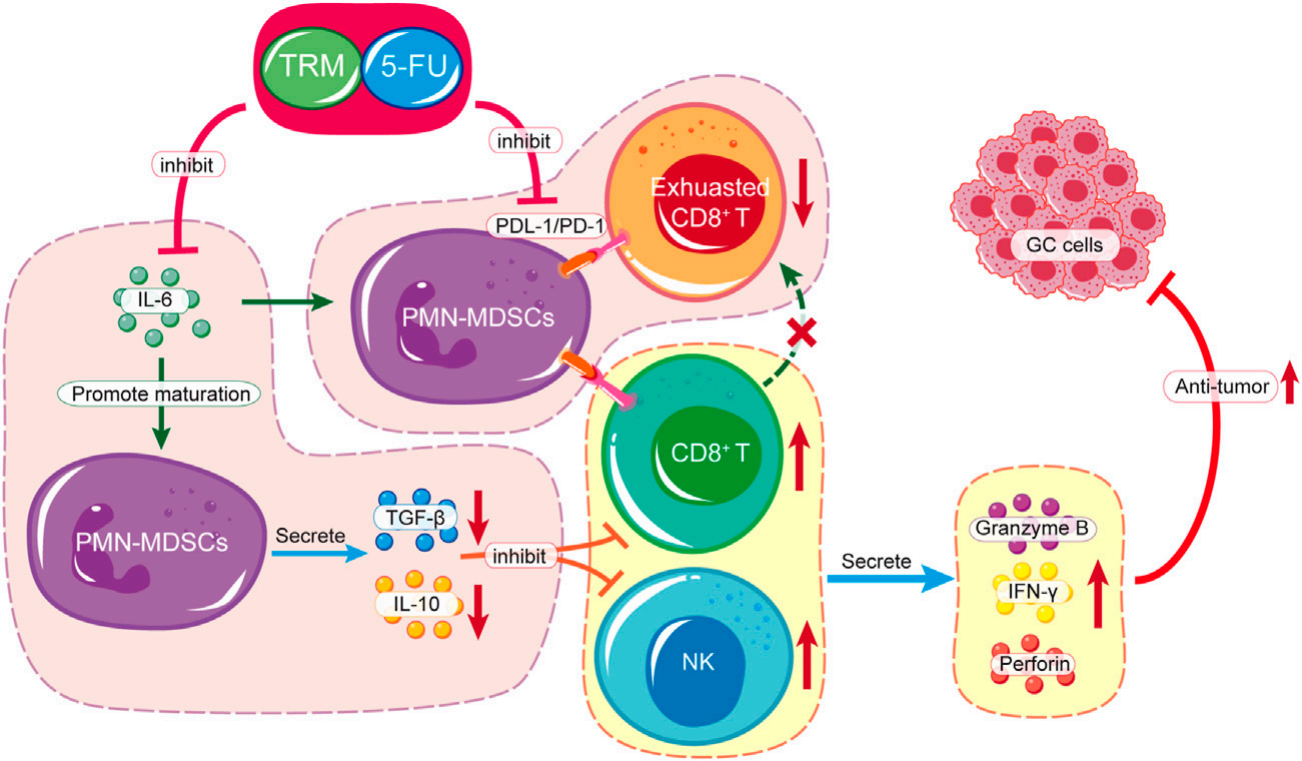
Schematic diagram of TRMBE in reshaping tumor immune microenvironment, enhancing 5-FU chemosensitivity and inhibiting tumor growth, invasion and metastasis.

In this study, we found that the combination of TRMBE and 5-FU achieved remarkable effect in targeting GC tumor microenvironment. Considering the different modes of action of immunotherapeutic and chemotherapeutic drugs, this new combinational strategy provides a new prospective for comprehensive treatment of GC. First, TRMBE combined with 5-FU significantly reduced the progression of gastric cancer and the risk of liver metastasis, prolonging survival time. Second, TRMBE and 5-FU combined therapy regulated the blood cytokines (IL-6, IL-10, TGF-β, IFN-γ), immune cells in spleen (PMN-MDSCs), and modulated the tumor microenvironment, most notably the percentage of infiltrating PMN-MDSCs, NK cells, and PD-1^+^ CD8^+^ T cells. Third, the depletion of CD8^+^ T cells or NK cells abolished the enhanced anticancer effect of the combined treatment, suggesting that these immune cells are indispensable for the combined treatment effect. The above results indicated that the PMN-MDSCs are the key cells targeted by the combination of TRMBE and 5-FU. The combined therapy regulated the cytokine IL-6 in peripheral blood, reducing the recruitment of PMN-MDSCs in TME. On the one hand, the reduced PMN-MDSCs produced less immunosuppressive factors (IL-10, TGF-β), resulting in the decreased inhibition of MDSCs on CTLs and NK cells. On the other hand, the reduction of PMN-MDSCs reduced the expression of PD-1 on CD8^+^ T cells, therefore increasing the cytotoxicity of CTLs. In addition, the combined therapy increased the percentage of NK cells by reducing the PMN-MDSCs level in the TME, thus enhancing the anti-tumor effect of 5-FU (Figure 7). However, there was no direct evidence showing that the PMN-MDSCs are upstream cells of CTLs and NK cells regulated by the combined treatment of TRMBE and 5-FU. This assumption warrants further study both *in vivo* or *in vitro* in the future.

In summary, the combination of TRMBE and 5-FU enhanced tumor cytotoxicity of 5-FU, reduced the metastasis of GC and prolonged survival time by inhibiting PMN-MDSCs and PD1^+^ CD8^+^ T cells, and stimulating NK cells in TME. Our findings provide a rational for the combination of TCMs with chemotherapy in GC therapy, and TRMBE is a promising candidate.

## Data Availability

The original contributions presented in the study are included in the article/Supplementary Materials, further inquiries can be directed to the corresponding authors.
