# Supplementary figures and images for: Trametes robiniophila Murr Sensitizes Gastric Cancer Cells to 5-Fluorouracil by Modulating Tumor Microenvironment
Source: Front Pharmacol. 2022 May 17;13:911663. doi: 10.3389/fphar.2022.911663 (PMC9152117; doi:10.3389/fphar.2022.911663)

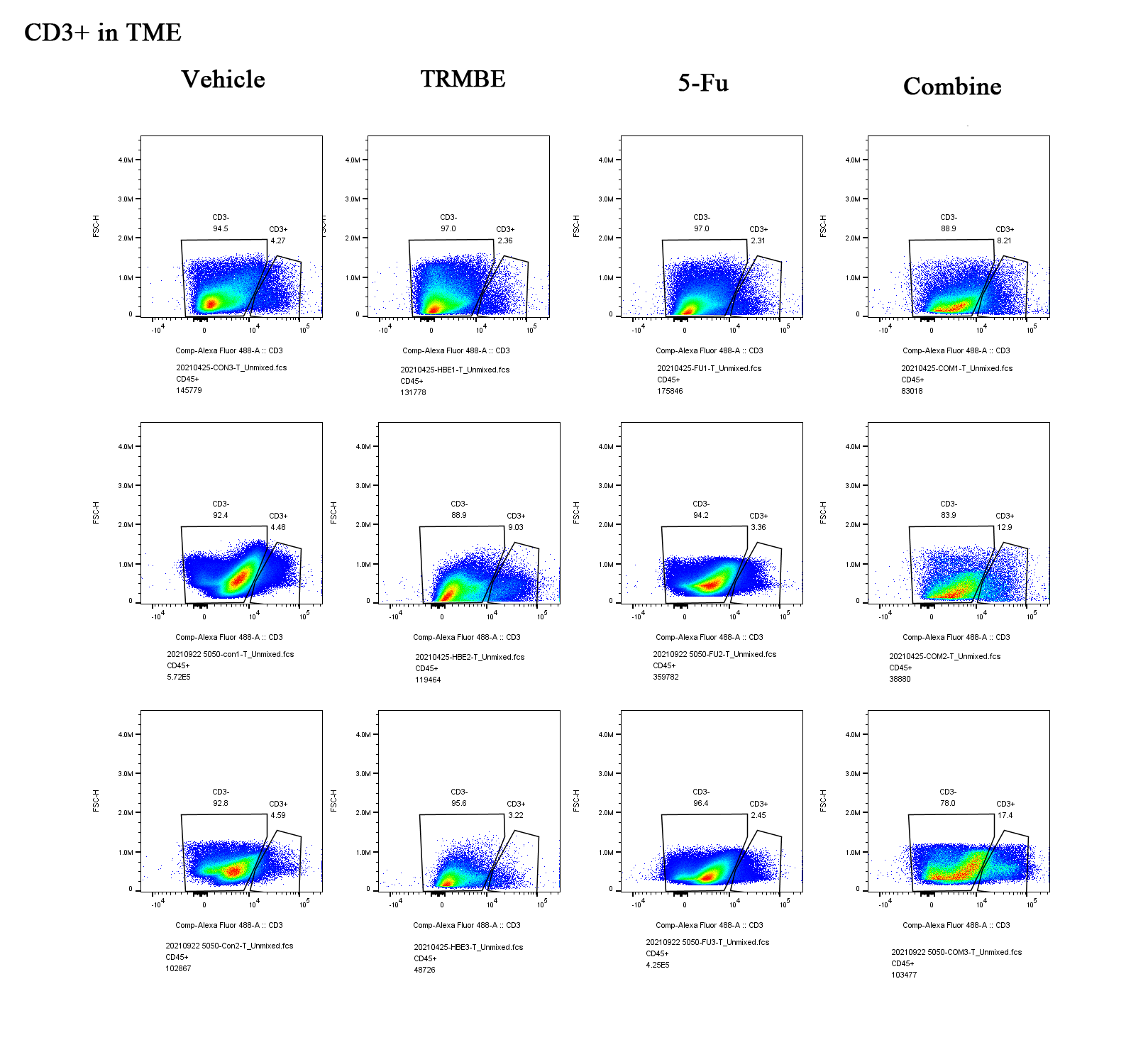

Supplement: Supplementary file 1 [file DataSheet2.zip › original data/flow cytometry/immune cells in TME/CD3+ IN TME.tif]

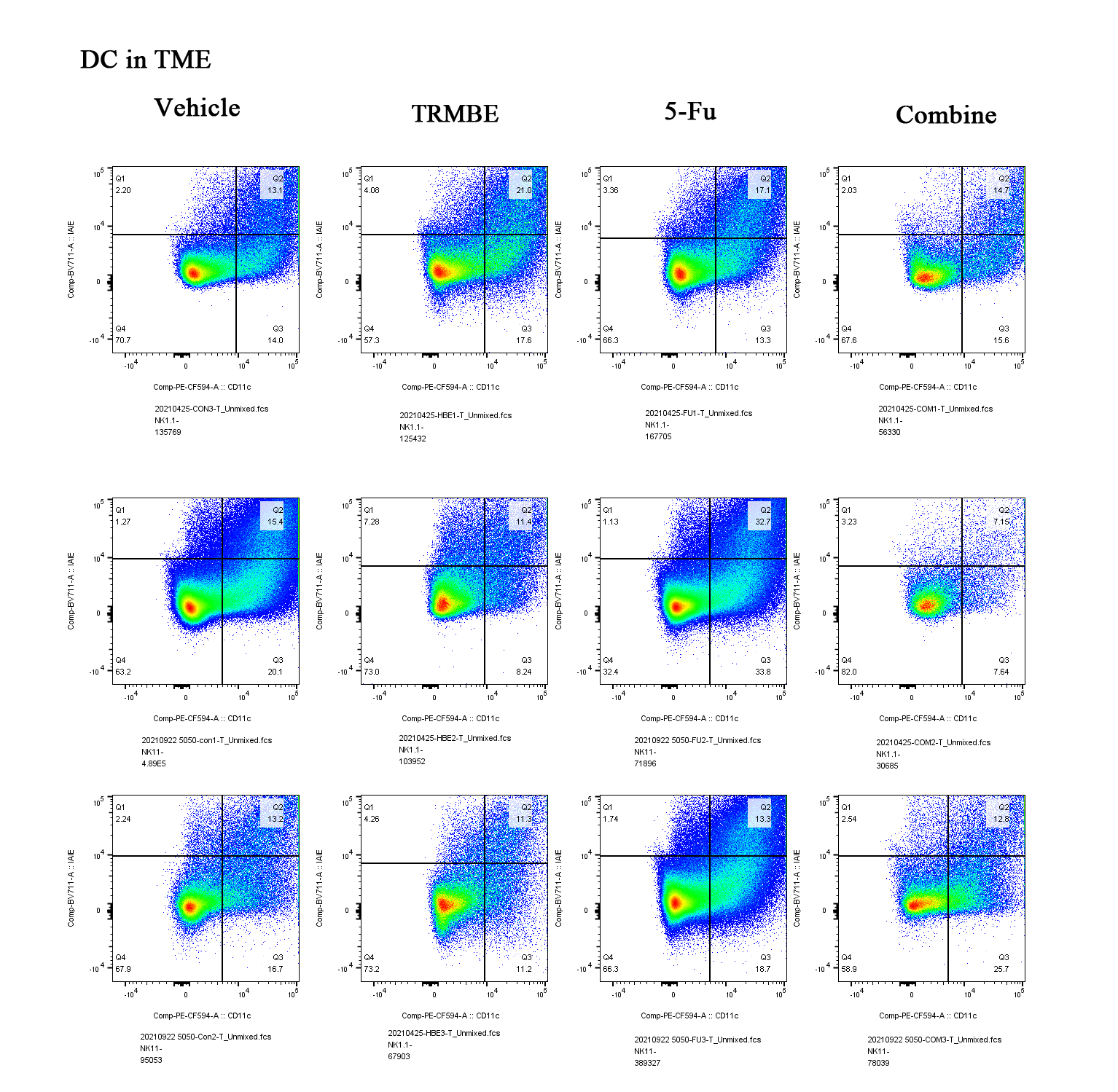

Supplement: Supplementary file 1 [file DataSheet2.zip › original data/flow cytometry/immune cells in TME/DC in TME.tif]

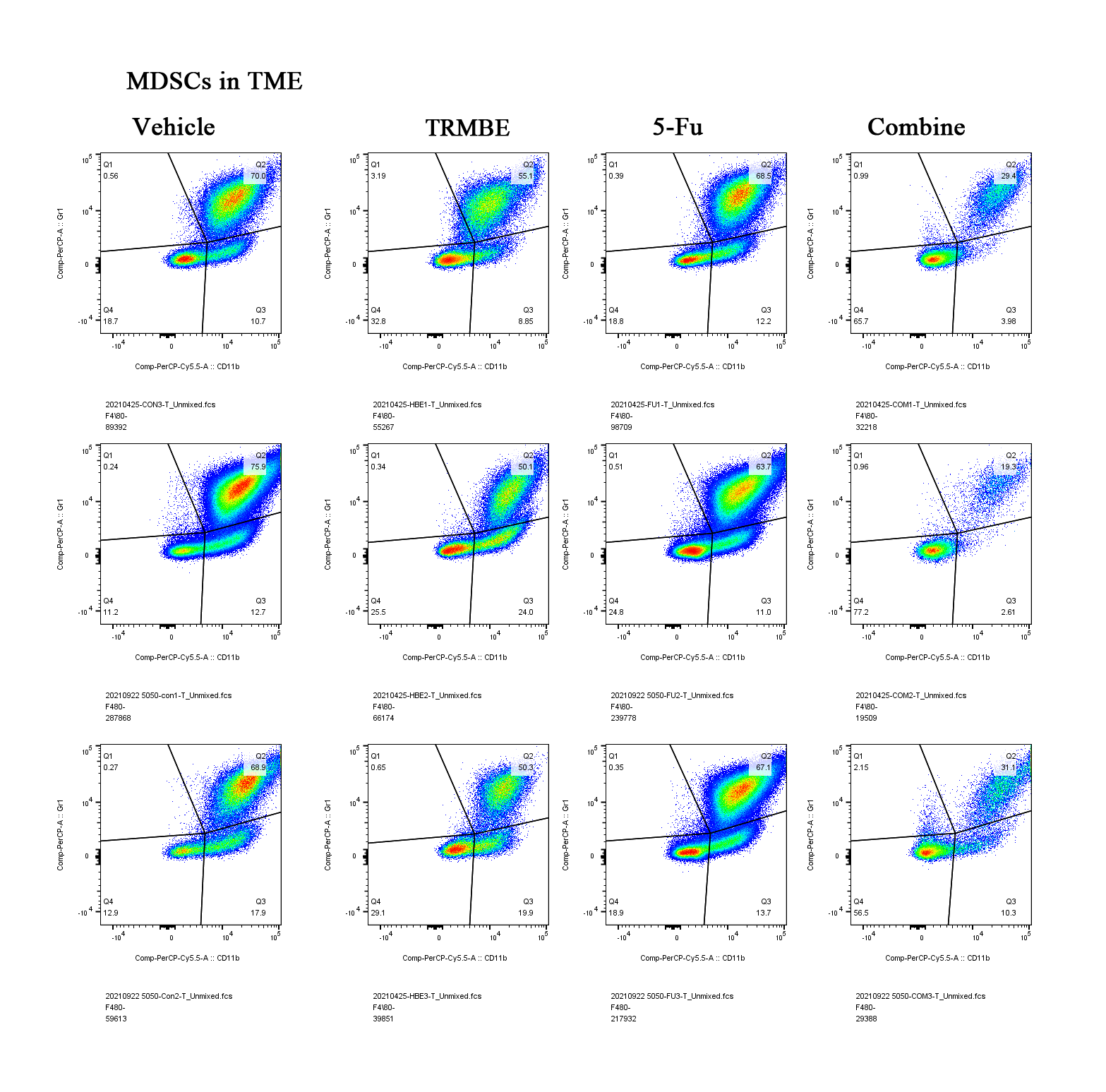

Supplement: Supplementary file 1 [file DataSheet2.zip › original data/flow cytometry/immune cells in TME/MDSCs in TME.tif]

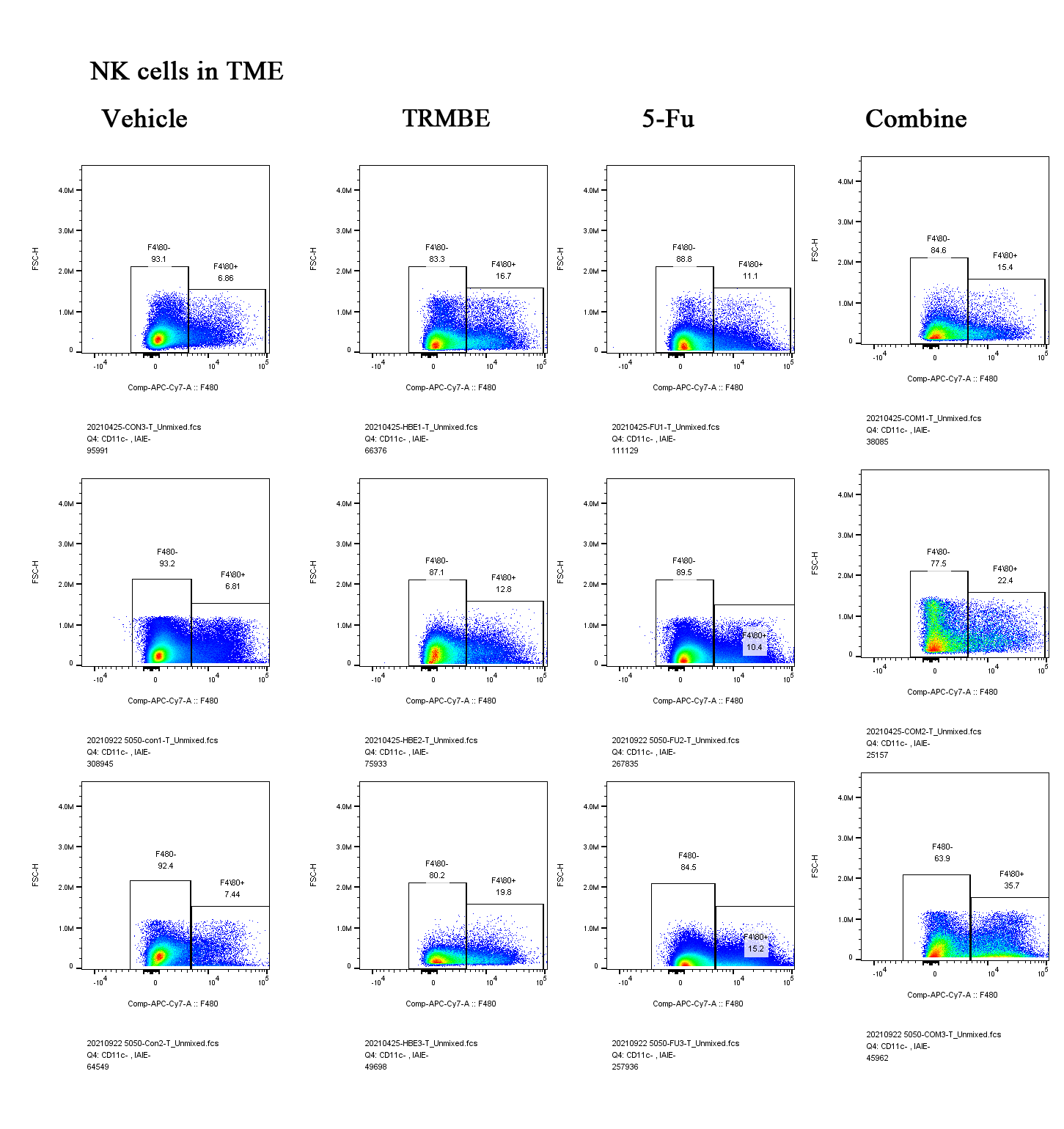

Supplement: Supplementary file 1 [file DataSheet2.zip › original data/flow cytometry/immune cells in TME/Macro IN TME.tif]

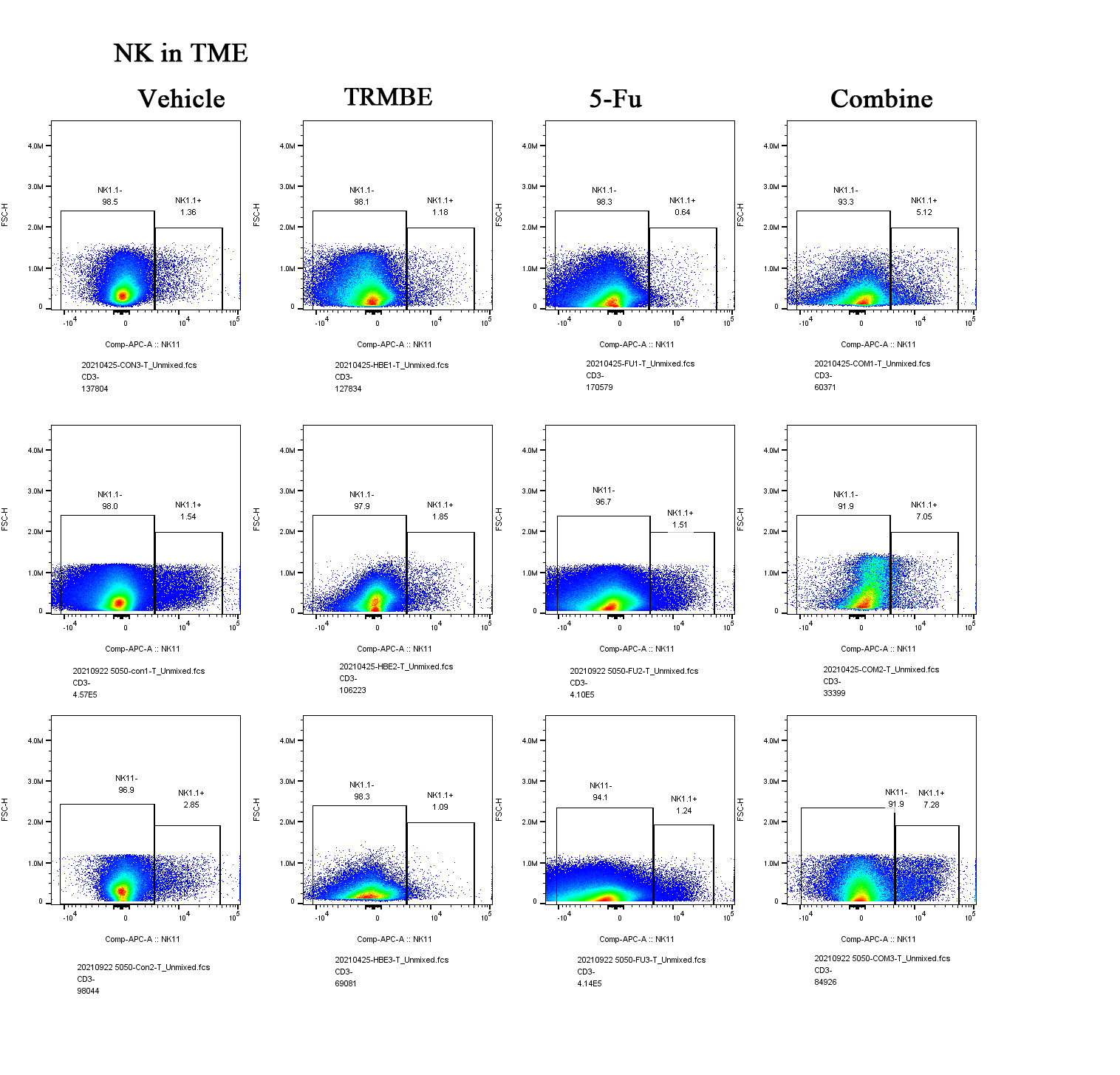

Supplement: Supplementary file 1 [file DataSheet2.zip › original data/flow cytometry/immune cells in TME/NK in TME.tif]

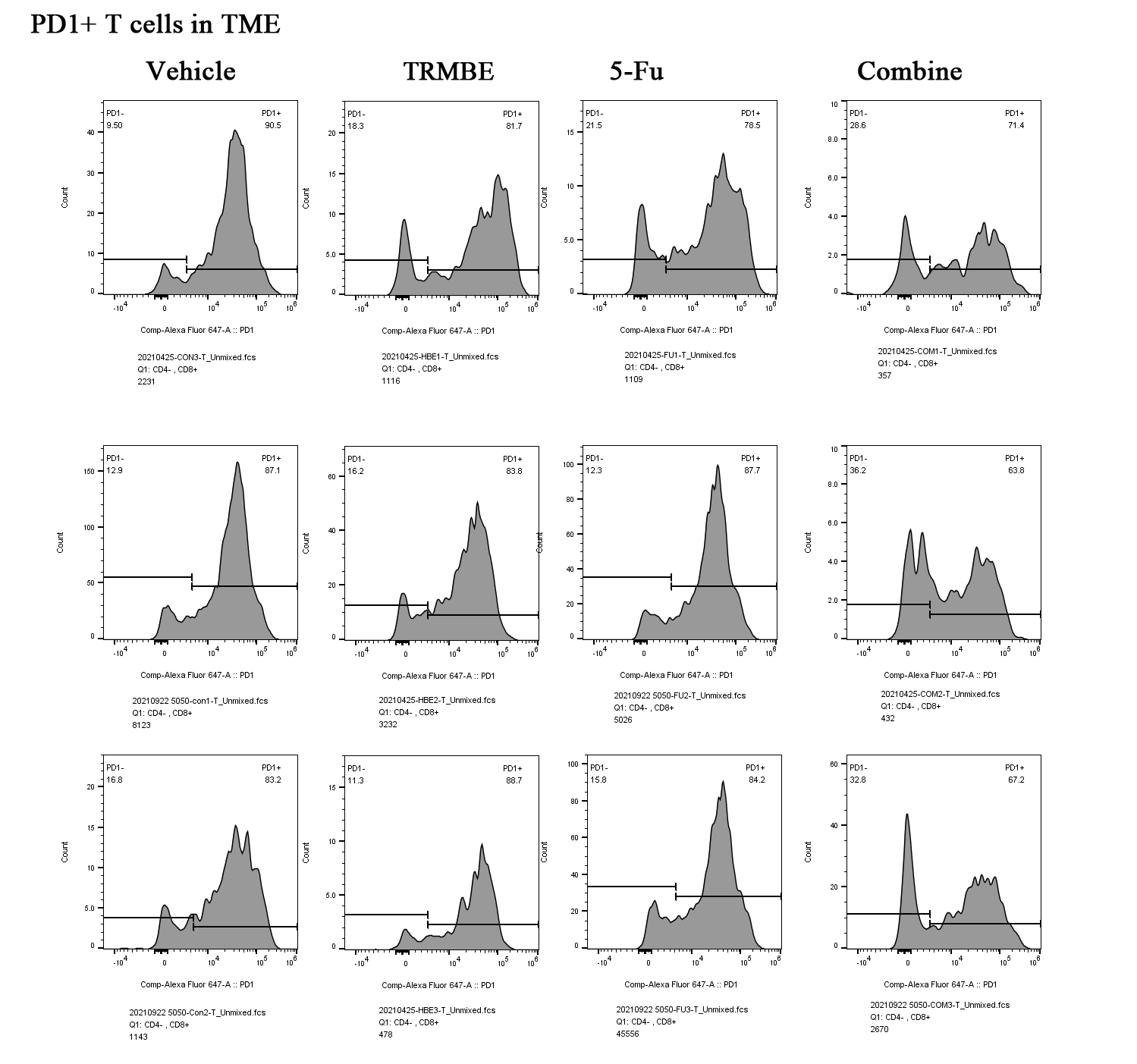

Supplement: Supplementary file 1 [file DataSheet2.zip › original data/flow cytometry/immune cells in TME/PD1+ T cells in TME.tif]

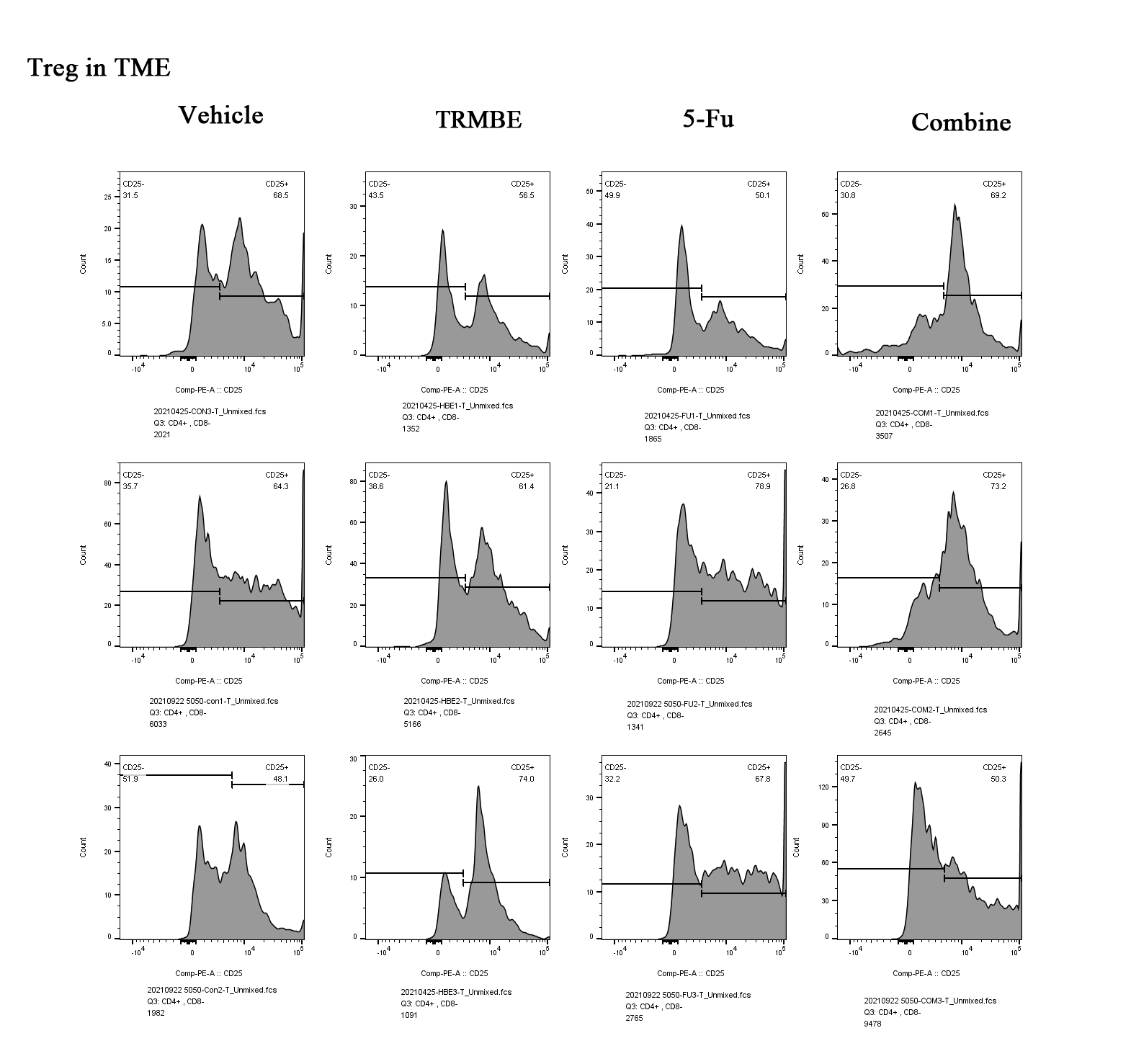

Supplement: Supplementary file 1 [file DataSheet2.zip › original data/flow cytometry/immune cells in TME/Treg in TME.tif]

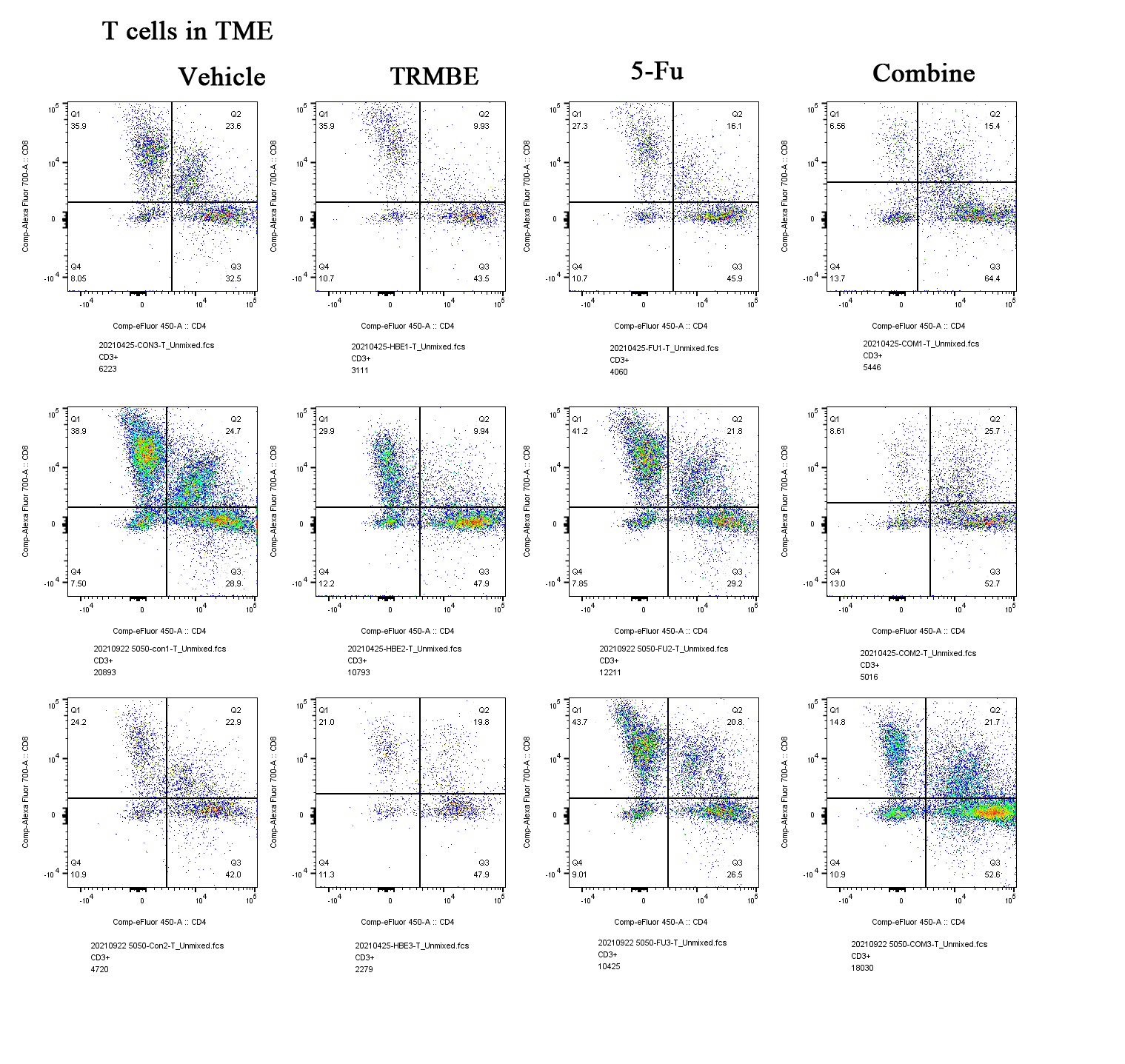

Supplement: Supplementary file 1 [file DataSheet2.zip › original data/flow cytometry/immune cells in TME/t cells in tme.tif]

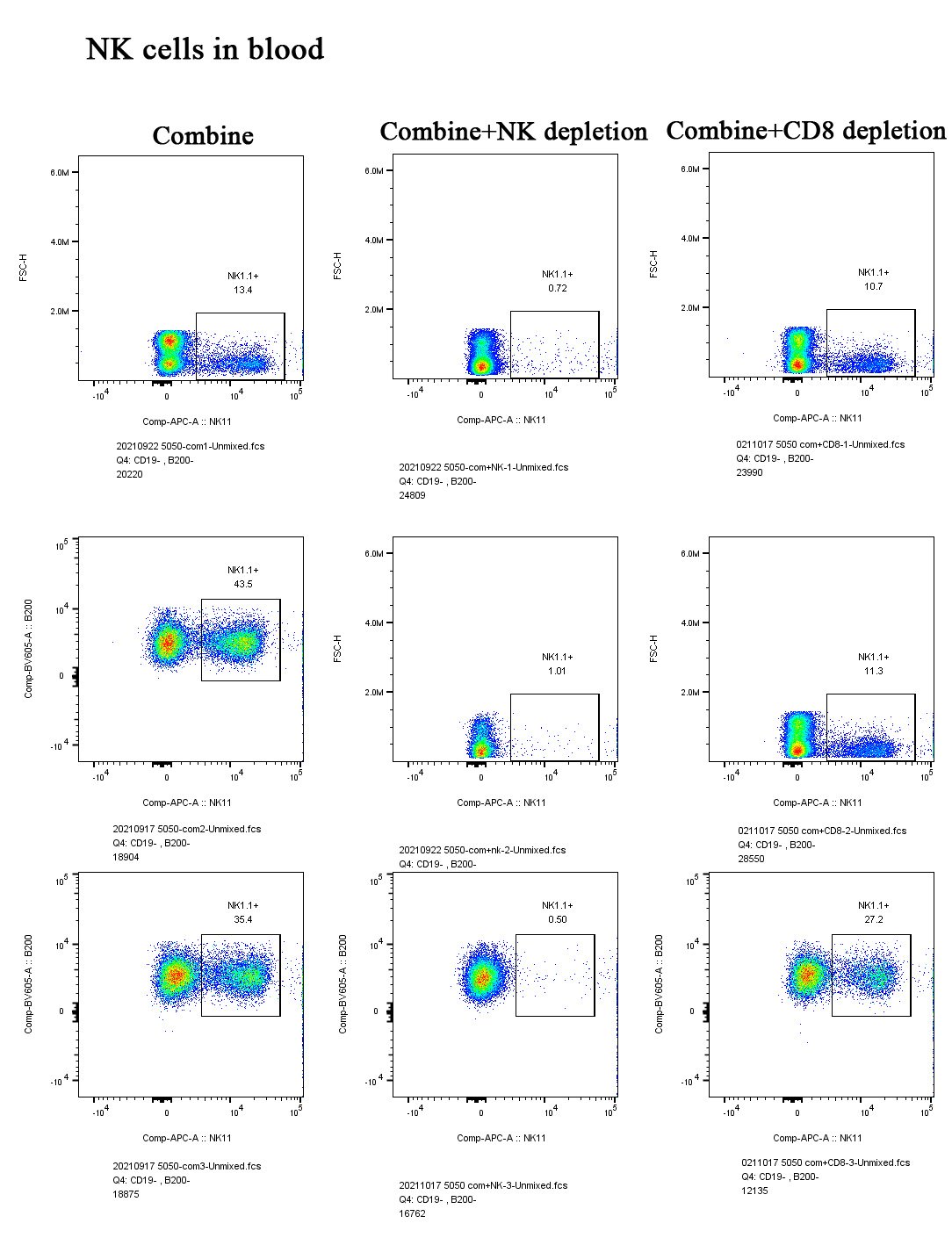

Supplement: Supplementary file 1 [file DataSheet2.zip › original data/flow cytometry/immune cells in immune cell depletion group/NK cells in blood.tif]

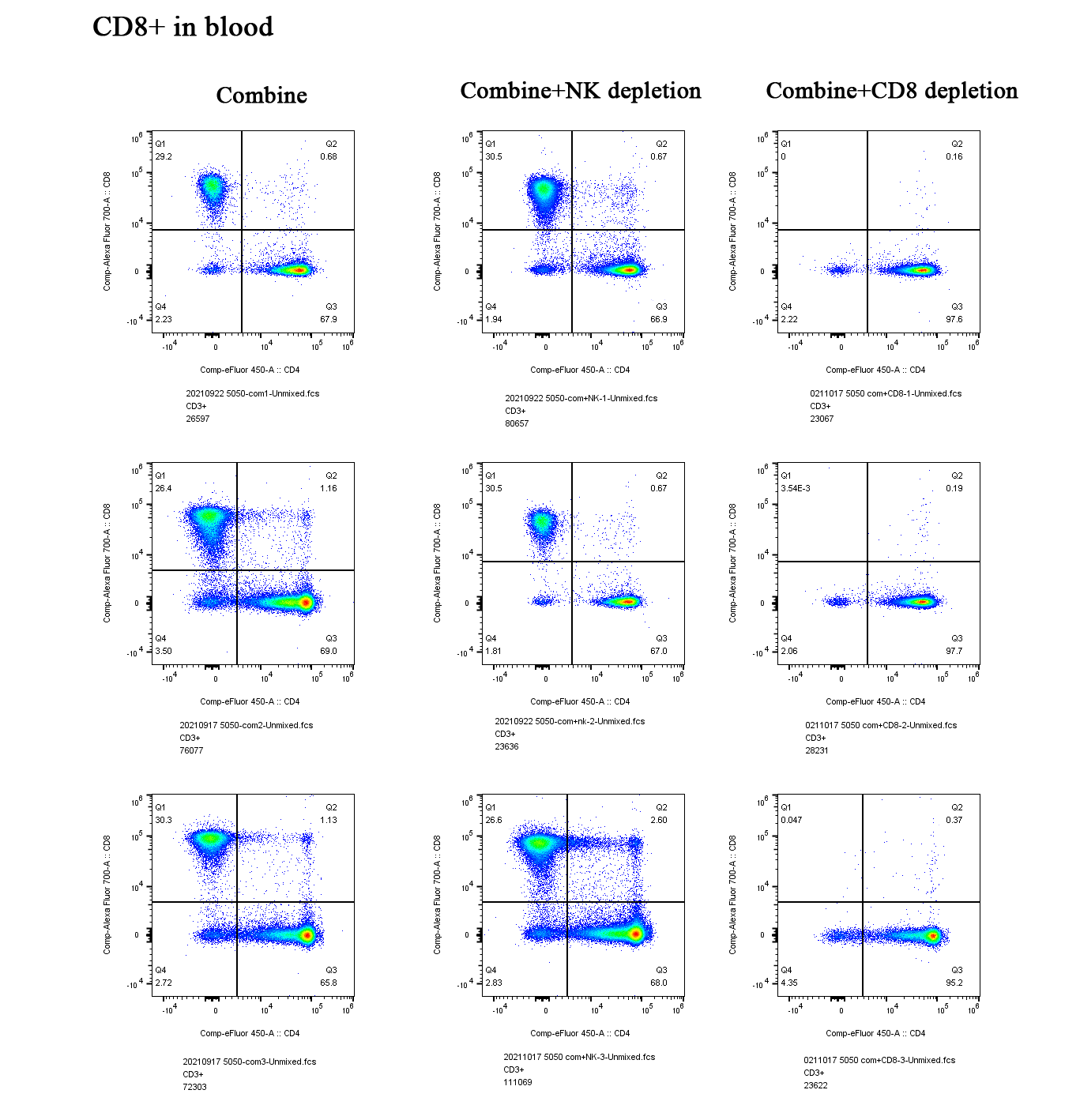

Supplement: Supplementary file 1 [file DataSheet2.zip › original data/flow cytometry/immune cells in immune cell depletion group/cd8+ in blood.tif]

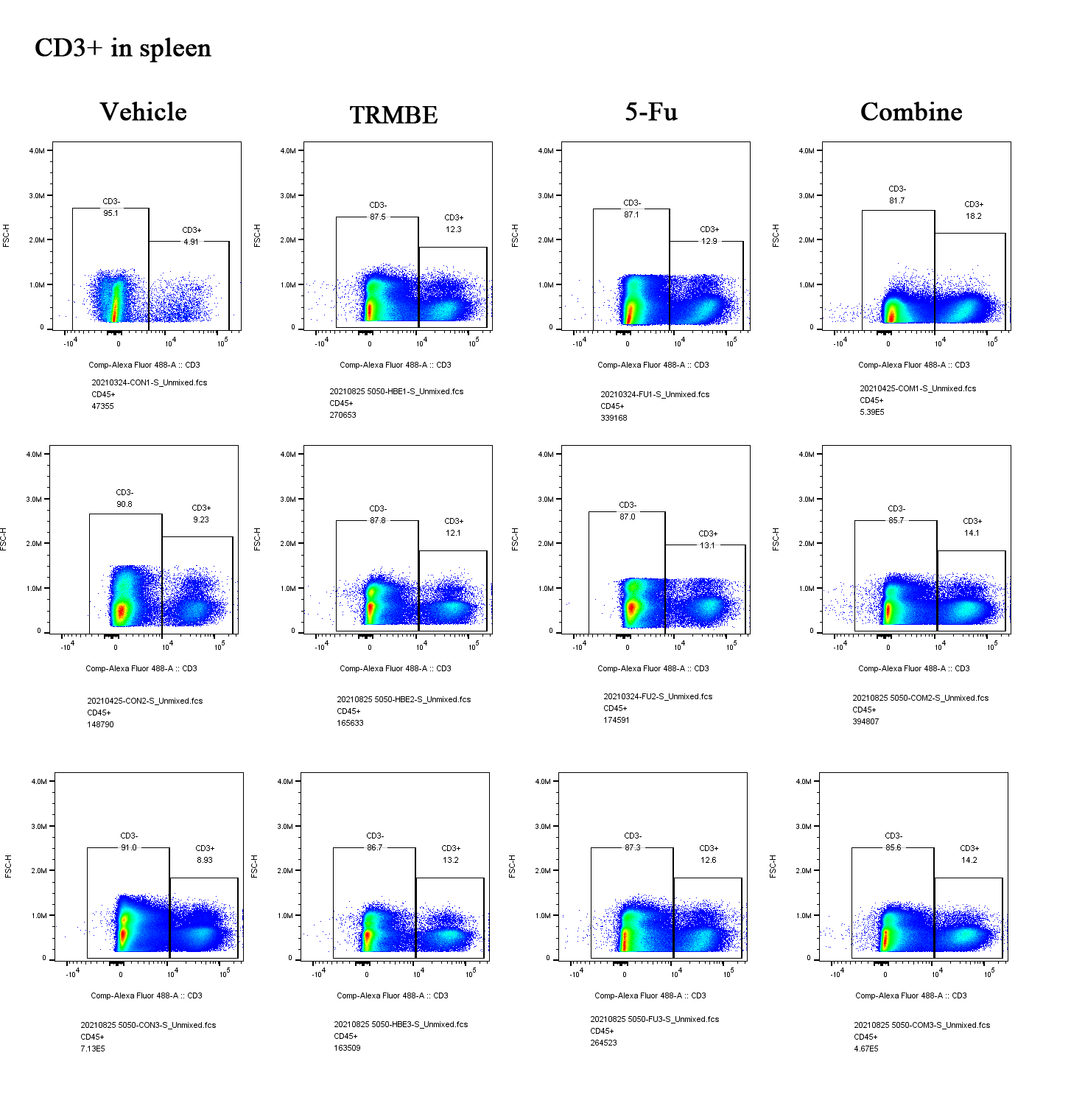

Supplement: Supplementary file 1 [file DataSheet2.zip › original data/flow cytometry/immune cells in spleen/CD3 in spleen.tif]

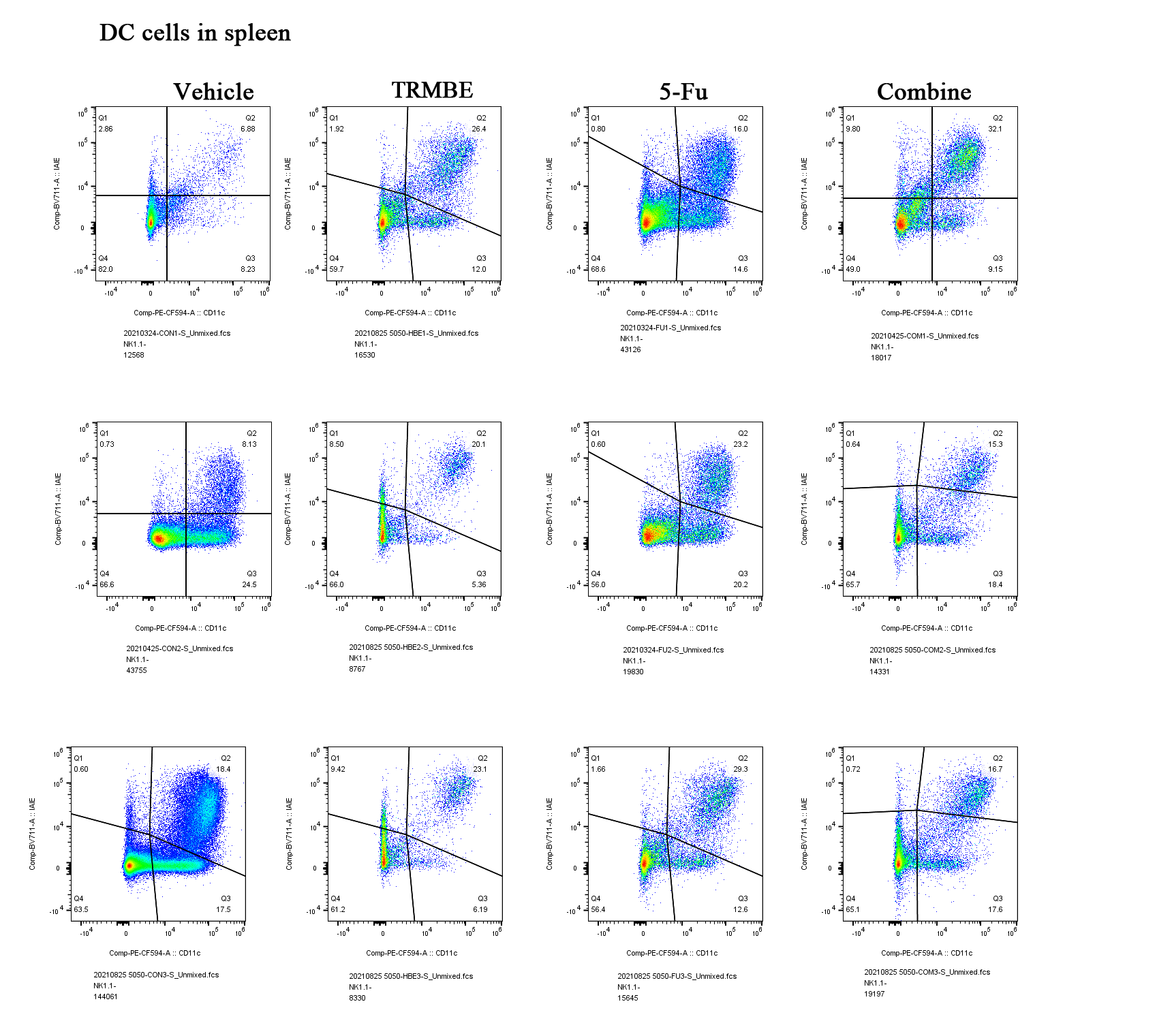

Supplement: Supplementary file 1 [file DataSheet2.zip › original data/flow cytometry/immune cells in spleen/DC cells in spleen.tif]

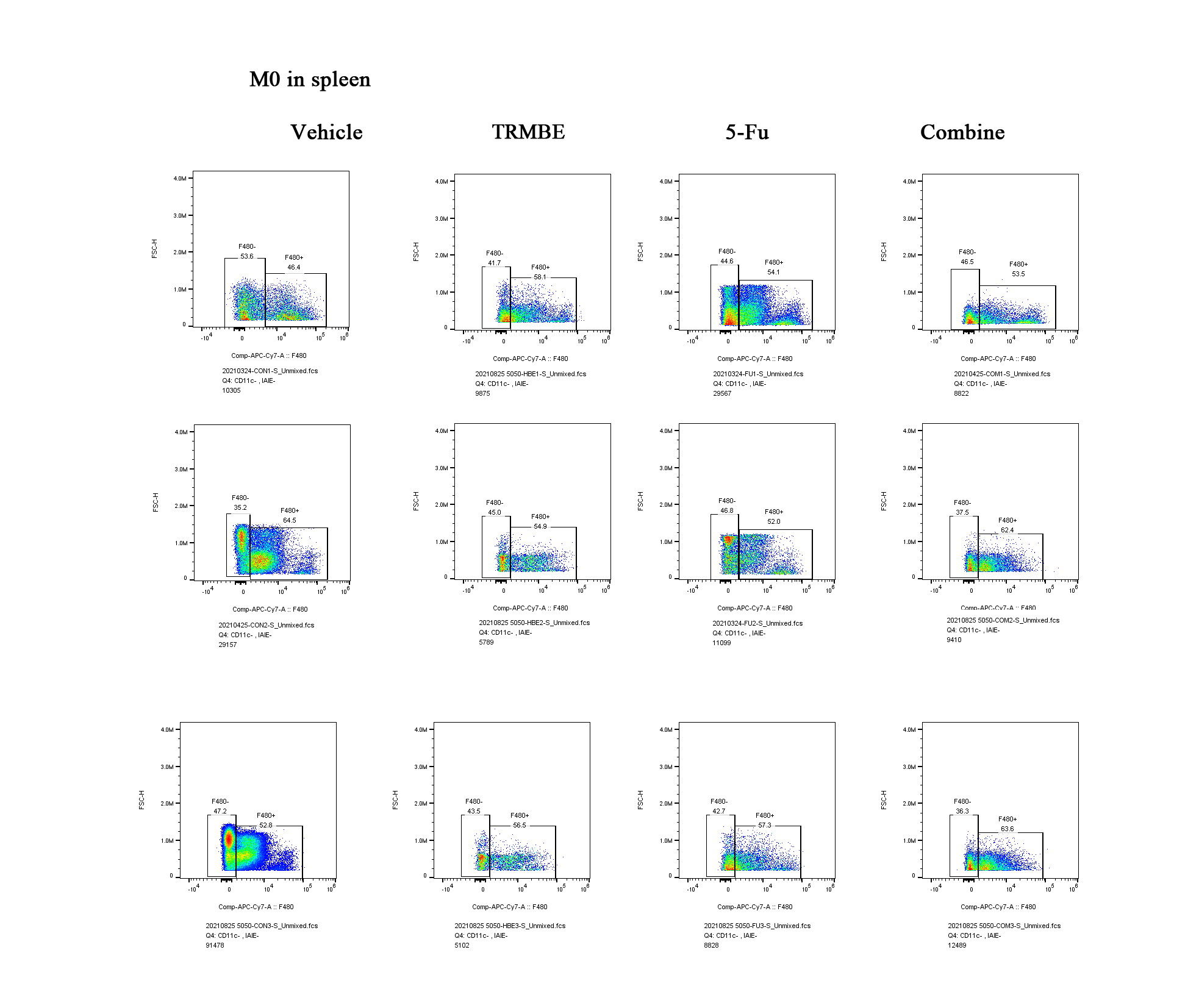

Supplement: Supplementary file 1 [file DataSheet2.zip › original data/flow cytometry/immune cells in spleen/M0 in spleen.tif]

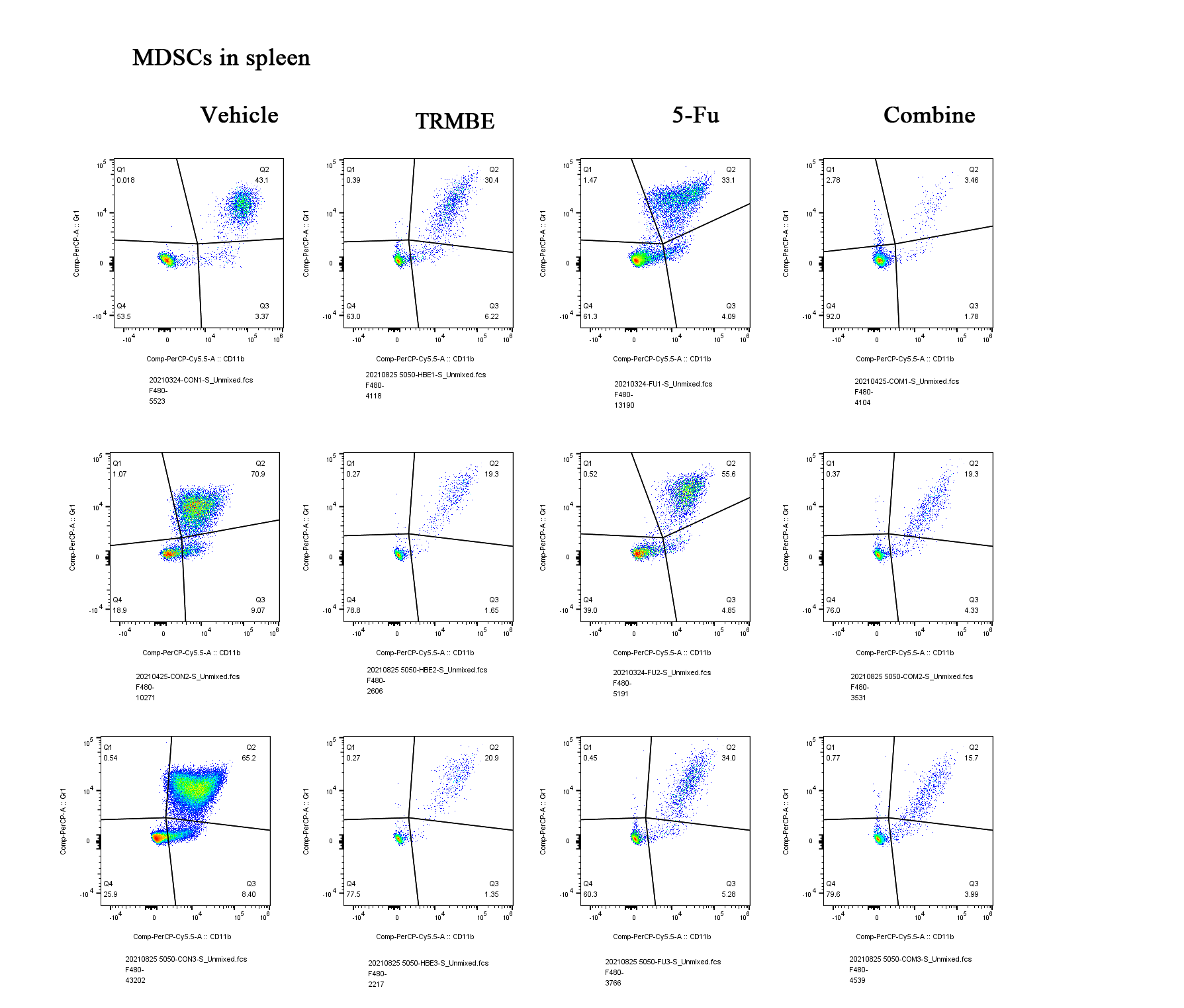

Supplement: Supplementary file 1 [file DataSheet2.zip › original data/flow cytometry/immune cells in spleen/MDSCs in spleen.tif]

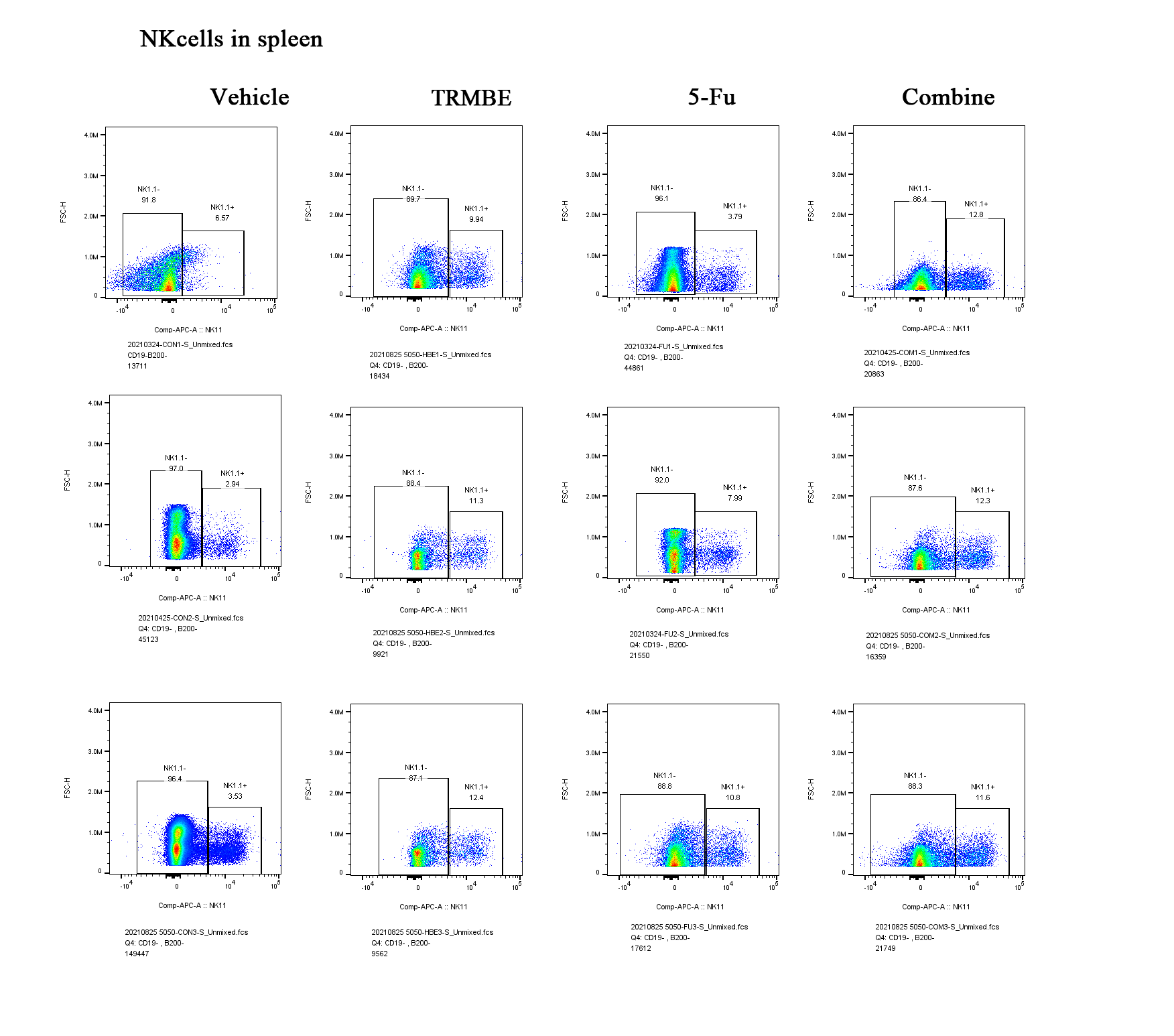

Supplement: Supplementary file 1 [file DataSheet2.zip › original data/flow cytometry/immune cells in spleen/NKcells in spleen.tif]

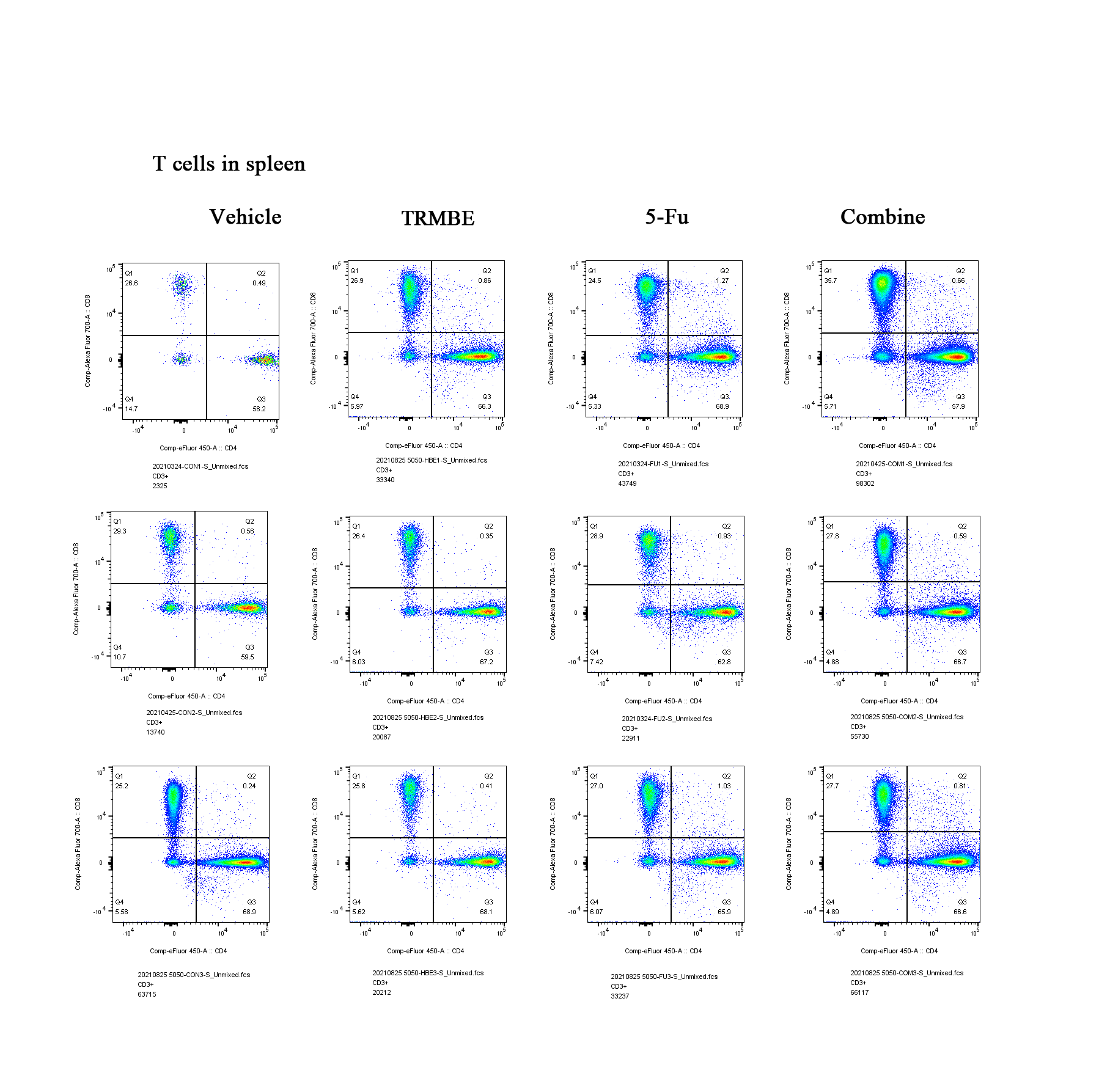

Supplement: Supplementary file 1 [file DataSheet2.zip › original data/flow cytometry/immune cells in spleen/T cells in spleen.tif]

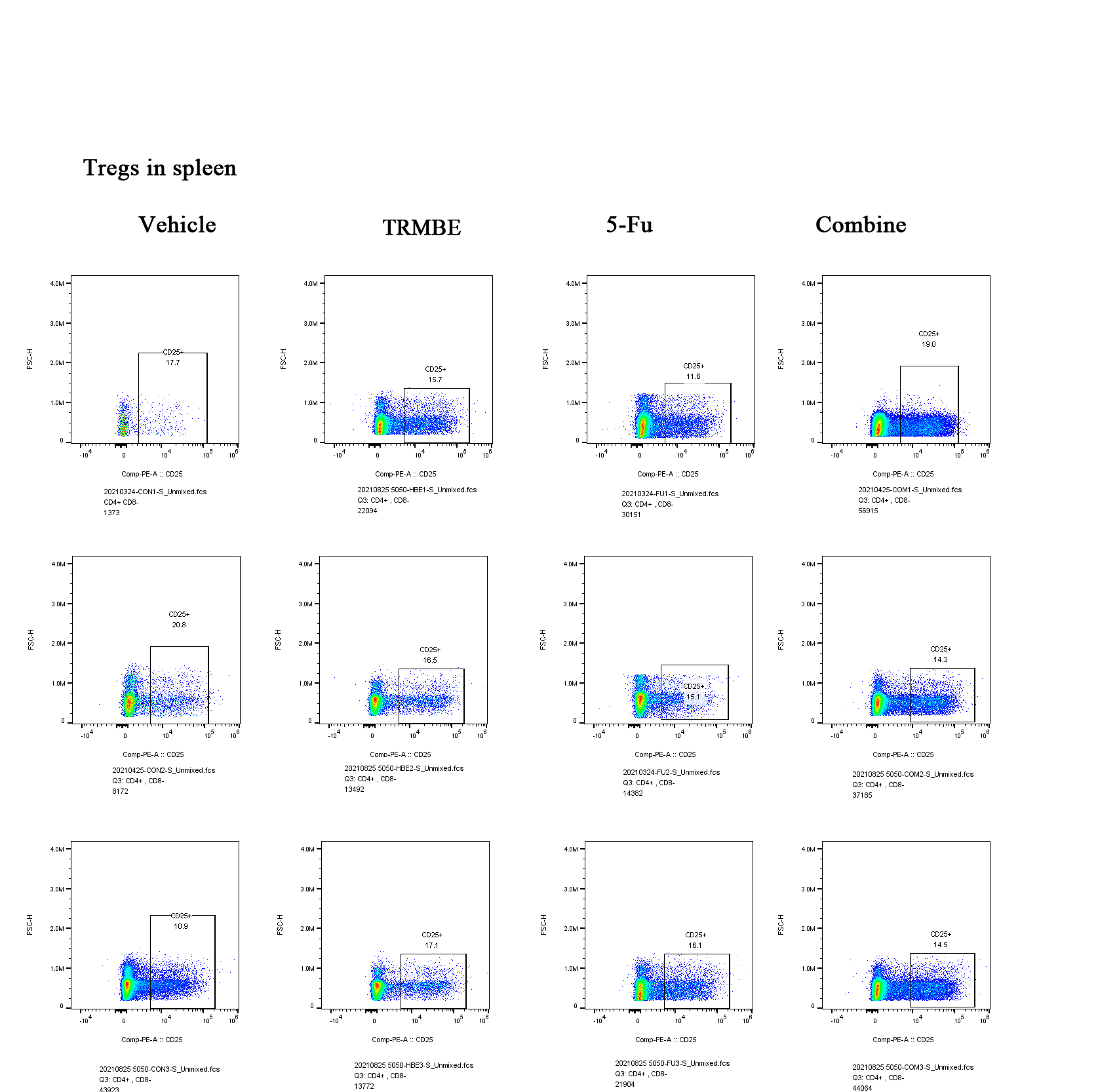

Supplement: Supplementary file 1 [file DataSheet2.zip › original data/flow cytometry/immune cells in spleen/Tregs in spleen.tif]

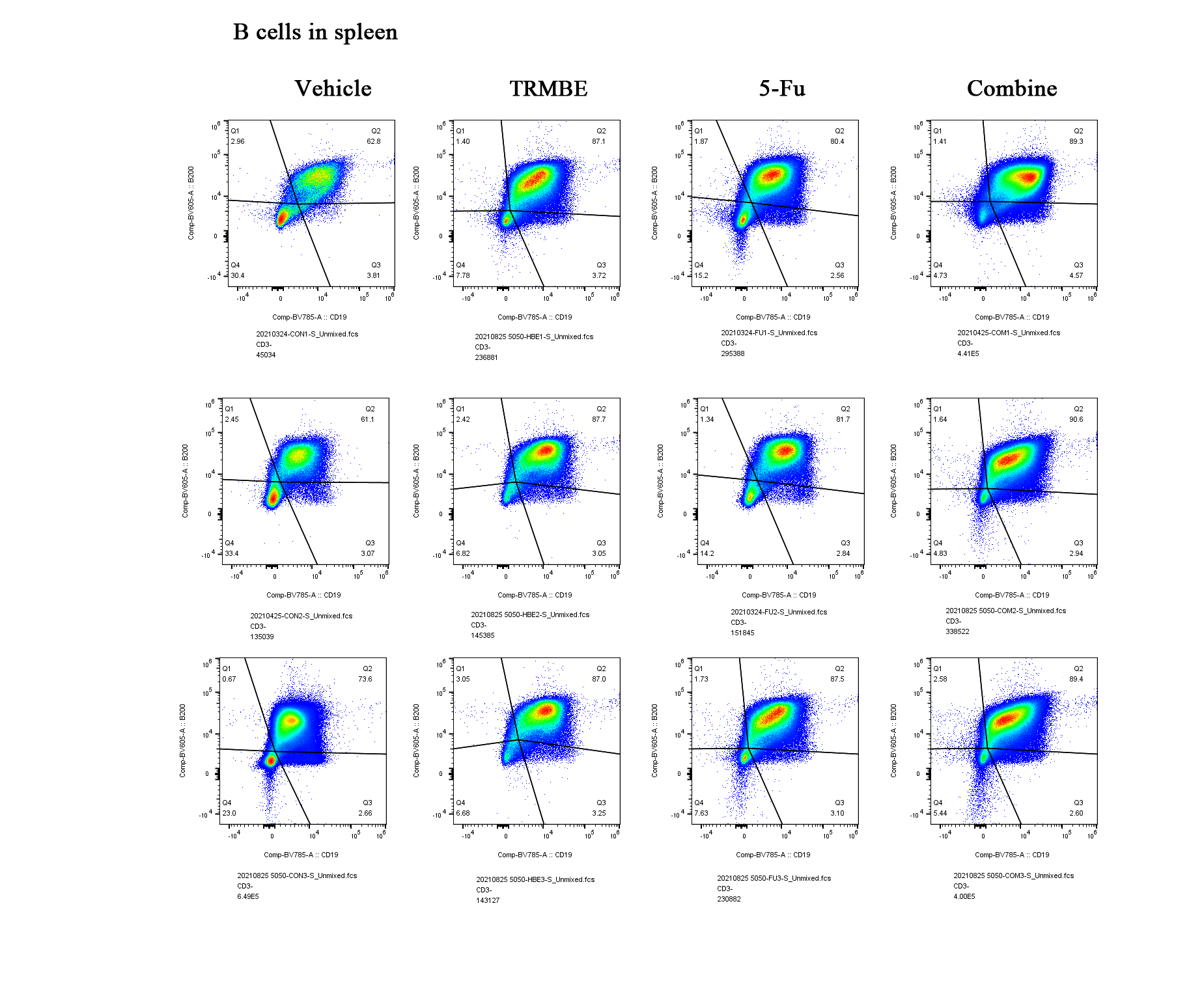

Supplement: Supplementary file 1 [file DataSheet2.zip › original data/flow cytometry/immune cells in spleen/b cells in spleen.tif]
