# Supplementary material for: Trametes robiniophila Murr Sensitizes Gastric Cancer Cells to 5-Fluorouracil by Modulating Tumor Microenvironment
Source: Front Pharmacol. 2022 May 17;13:911663. doi: 10.3389/fphar.2022.911663 (PMC9152117; doi:10.3389/fphar.2022.911663)
Supplement: Supplementary file 2 [file DataSheet1.docx]

Supplementary Material

**The main components of Trametes robiniophila Murr n-butanol Extract by LC-MS**

# Supplementary Data

**1.1 Treatment of Trametes robiniophila Murr n-butanol Extract**

1mg Trametes robiniophila Murr n-butanol Extract was added with 1ml of precooled methanol, vibrated for 20min, centrifuged with 10000g at 4 ℃ for 10min, 600ul of which was lyophilized, and then dissolved in 100ul methanol/water(4:1), and 4ul of supernatant was taken for experiment.

**1.2 Chromatographic conditions**

Chromatographic column: waters UPLC BEH C18(1.8um 2.1mm*50mm).

mobile phase: A (0.1% formic acid in water) and B (acetonitrile). Elution procedure: 2% B was maintained for 1 min, then increased to 80% within 24 min, and reached 100% at 26 min, after 4 min, the column was balanced for 5 min. The flow rate was 0.35 ml/min, the injection volume was 4ul, the equilibrium time was 5 min before each injection, and the column temperature was 50 ℃.

**1.3 Mass spectrometry identification**

Positive ion detection mode ESI ionization mode was adopted, and the mass scanning range was 80-1000m/Z. Nitrogen was used in various gas paths. Mass spectrum parameters were shown in the following table.

| Survey Ion mode & Polarity: | ESI+ |
| --- | --- |
| Mass: | 80-1000M/Z |
| Capillary: | 3000V |
| Sampling Cone : | 35V |
| Desolvation Temperature: | 300℃ |
| Desolvation Gas Flow: | 500L/H |
| Cone Gas Flow: | 50L/H |
| Source Temperature: | 100℃ |
| Signal to Noise Ratio | 5 |

# Supplementary Figures and Tables

## Supplementary Figures

*2.1.1 BPC Chromatogram*

**Supplementary Figure 1.** BPC Chromatogram

*2.1.2 Mass spectrum peak and compound after normalization*

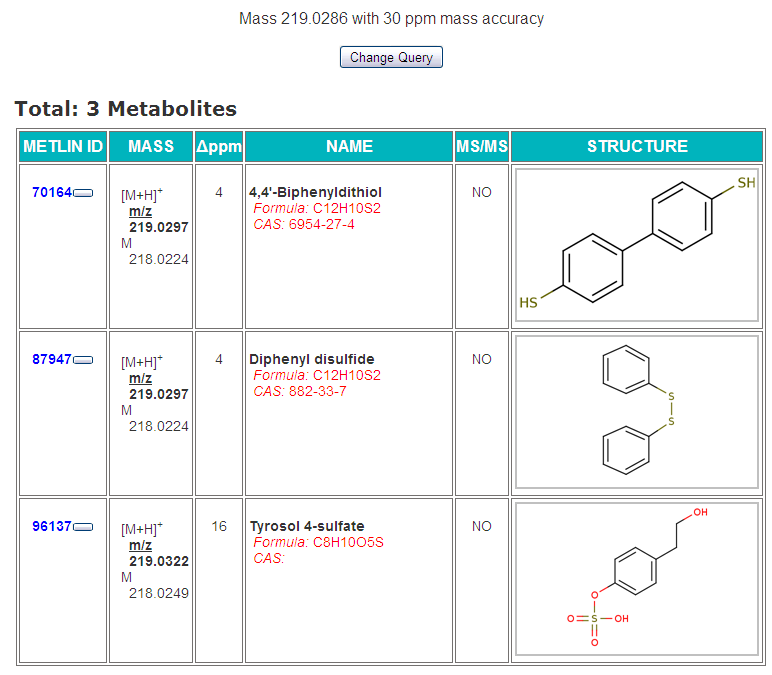


**Supplementary Figure 2.** Mass 219.0286 with 30 ppm mass accuracy

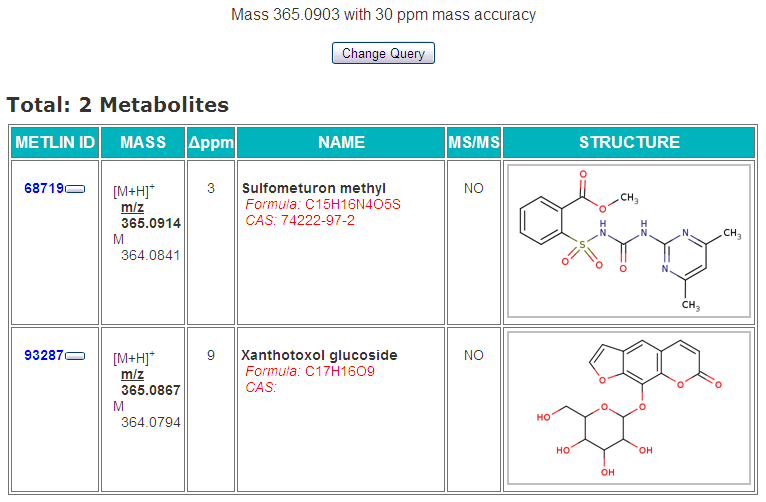


**Supplementary Figure 3.** Mass 365.0903 with 30 ppm mass accuracy

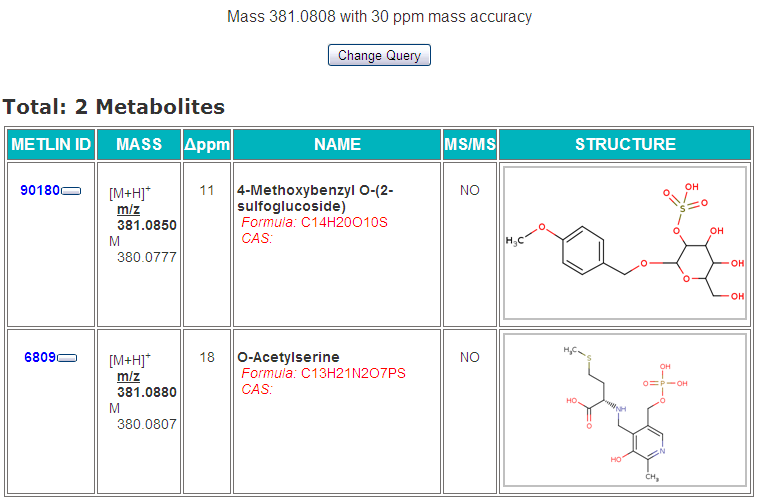


**Supplementary Figure 4.** Mass 381.0808 with 30 ppm mass accuracy

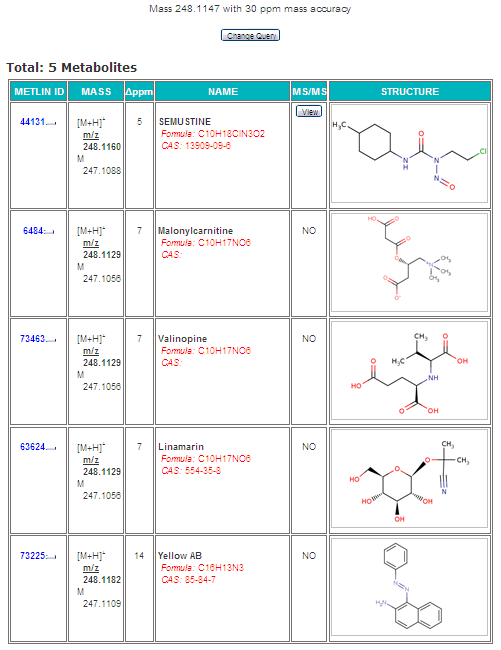


**Supplementary Figure 5.** Mass 248.1147 with 30 ppm mass accuracy

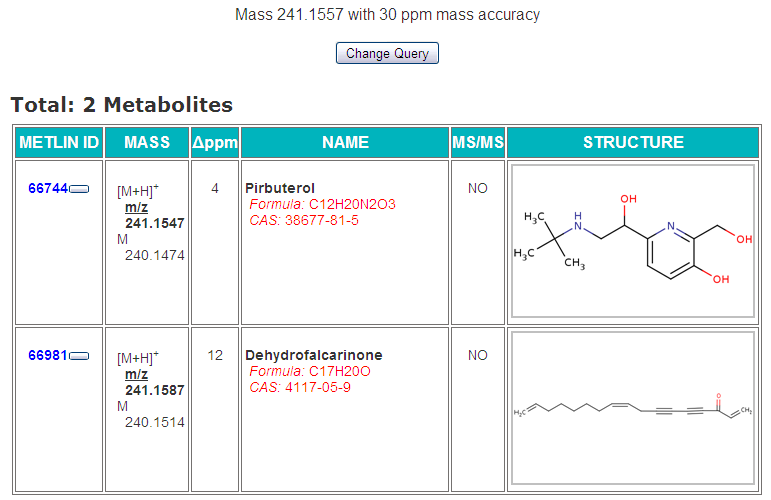


**Supplementary Figure 6.** Mass 241.1557 with 30 ppm mass accuracy

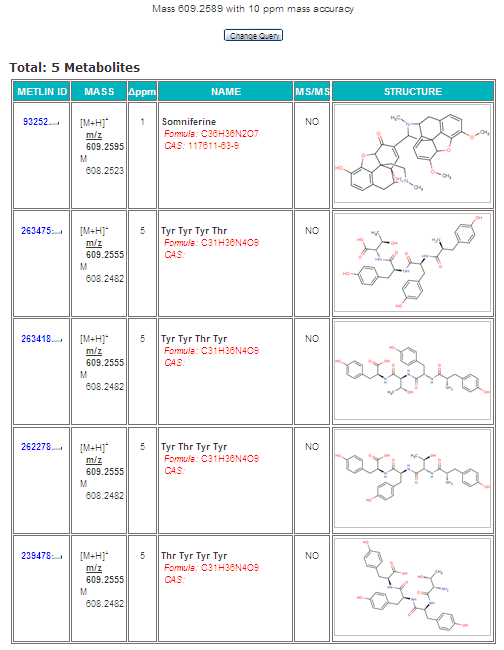


**Supplementary Figure 7.** Mass 609.2589 with 30 ppm mass accuracy

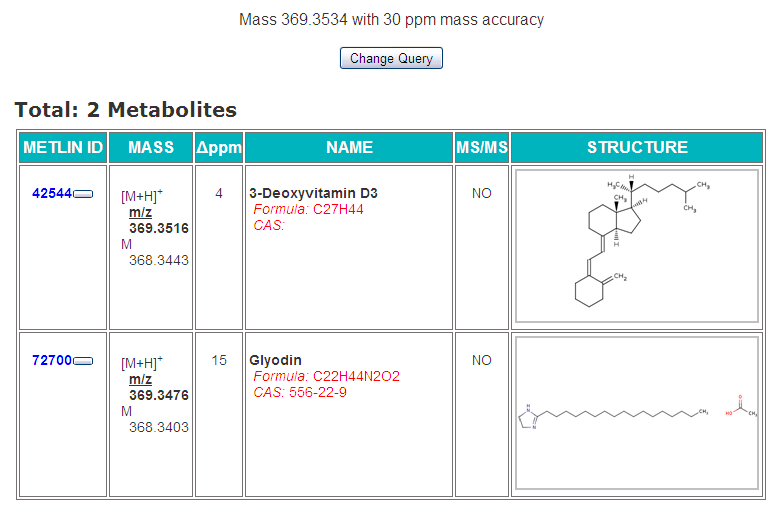


**Supplementary Figure 8.** Mass 369.3534 with 30 ppm mass accuracy

## Supplementary Tables

| No. | Metlin ID | Molecular formula | Potential substance |
| --- | --- | --- | --- |
| 1 | 70164 | C12H10S2 | 4,4'-Biphenyldithiol |
| 2 | 87947 | C12H10S2 | Diphenyl disulfide |
| 3 | 96137 | C8H10O5S | Tyrosol 4-sulfate |
| 4 | 68719 | C15H16N4O5S | Sulfometuron-Methyl |
| 5 | 93287 | C17H16O9 | Xabthotoxol glucoside |
| 6 | 90180 | C14H20O10S | 4-Methoxybenzyl O-(2-sulfoglucoside) |
| 7 | 6809 | C13H21N2O7PS | O-Acetylserine |
| 8 | 44131 | C10H18CIN3O2 | Semustine |
| 9 | 6484 | C10H17NO6 | Malonylcarnitine |
| 10 | 73463 | C10H17NO6 | Valinopine |
| 11 | 63624 | C10H17NO6 | Linamarin |
| 12 | 73225 | C16H13N3 | Yellow AB |
| 13 | 66744 | C12H20N2O3 | Pirbuterol |
| 14 | 66981 | C17H20O | Dehydrofalcarinone |
| 15 | 93252 | C36H36N2O7 | Somniferine |
| 16 | 263475 | C31H36N4O9 | Tyr Tyr Tyr Thr |
| 17 | 263418 | C31H36N4O9 | Tyr Tyr Thr Tyr |
| 18 | 262278 | C31H36N4O9 | Tyr Thr Tyr Tyr |
| 19 | 239478 | C31H36N4O9 | Thr Tyr Tyr Tyr |
| 20 | 42544 | C27H44 | 3-Deoxyvitamin D3 |
| 21 | 72700 | C22H44N2O2 | Glyodin |

**Supplementary Table 1.** The LC-MS Component Analysis of Trametes robiniophila Murr n-butanol Extract
