# Supplementary material for: Trametes robiniophila Murr Sensitizes Gastric Cancer Cells to 5-Fluorouracil by Modulating Tumor Microenvironment
Source: Front Pharmacol. 2022 May 17;13:911663. doi: 10.3389/fphar.2022.911663 (PMC9152117; doi:10.3389/fphar.2022.911663)
Supplement: Supplementary file 1 [file DataSheet2.zip › original data/Original data materials.doc]

**Original Data**

1.3 **The data of subcutaneous GC mice.**

**The tumor long and short diameters of subcutaneous GC mice in four therapy groups (mm).**

| Group | No. | Day 3 | | Day 4 | | Day 5 | | Day 6 | | Day 7 | | Day 8 | | Day 9 | | Day 10 | | Day 11 | | Day 12 | | Day 13 | |
| --- | --- | --- | --- | --- | --- | --- | --- | --- | --- | --- | --- | --- | --- | --- | --- | --- | --- | --- | --- | --- | --- | --- | --- |
|  |  | LD | SD | LD | SD | LD | SD | LD | SD | LD | SD | LD | SD | LD | SD | LD | SD | LD | SD | LD | SD | LD | SD |
| Vehicle | 1 | 4.96 | 4.14 | 5.62 | 3.99 | 5.79 | 5.05 | 7.59 | 5.77 | 8.84 | 6.09 | 10.9 | 6.98 | 11.7 | 8.86 | 12.4 | 9.6 | 13.6 | 11.7 | 13.68 | 12.5 | 15.32 | 13.16 |
|  | 2 | 4.89 | 3.1 | 3.52 | 3.47 | 5.98 | 4.12 | 6.94 | 4.88 | 9.09 | 6.03 | 12.54 | 7.96 | 11.7 | 9.05 | 13.6 | 8.9 | 13.7 | 9.95 | 15.7 | 13.5 | 16.29 | 13.66 |
|  | 7 | 3.68 | 2.92 | 4.3 | 3.76 | 5.05 | 4 | 7.93 | 5.4 | 8.06 | 5.3 | 9.57 | 6.52 | 10.14 | 8.23 | 9.12 | 7.6 | 10.34 | 8.28 | 12.41 | 10.68 | 12.6 | 10.74 |
|  | 8 | 3.36 | 2.94 | 6.13 | 4.26 | 4.4 | 4.45 | 5.63 | 4.66 | 6.39 | 5.92 | 8.22 | 7.38 | 8.88 | 7.96 | 8.32 | 7.25 | 14.4 | 10.3 | 14.43 | 12 | 16.9 | 11.49 |
|  | 10 | 3.4 | 2.52 | 5.75 | 4.63 | 7.31 | 4.56 | 8.6 | 6.16 | 10.08 | 5.72 | 12.76 | 7.46 | 12 | 6.18 | 13.24 | 8.1 | 14.3 | 8.08 | 13.81 | 10.24 | 16.56 | 10.6 |
|  | 12 | 2.68 | 2.12 | 3.92 | 3.03 | 4.77 | 3.5 | 7.71 | 4.73 | 8.47 | 6.47 | 6.4 | 6.27 | 10 | 6.66 | 11.39 | 7.56 | 15.2 | 9.6 | 16.4 | 11.2 | 18 | 11.3 |
| 5-FU | 16 | 4.87 | 3.51 | 5.5 | 4.03 | 5.39 | 5.27 | 6.6 | 6.46 | 6.36 | 6.33 | 7.02 | 7.02 | 8.1 | 8 | 8.59 | 8.2 | 9.83 | 8.86 | 10.11 | 10.06 | 10.62 | 9.5 |
|  | 18 | 4 | 3.33 | 4.17 | 3.77 | 6.12 | 4.72 | 6.05 | 4.79 | 6.27 | 5.21 | 8.28 | 5.67 | 8.29 | 5.4 | 9.43 | 5.9 | 10.97 | 8.1 | 11.41 | 8.3 | 13.35 | 9.5 |
|  | 22 | 3.13 | 3.1 | 4.95 | 3.58 | 4.66 | 3.69 | 4.72 | 3.68 | 4.92 | 3.87 | 9.39 | 7.35 | 11 | 6.28 | 10 | 7.25 | 11.1 | 7.7 | 11.03 | 8.32 | 11.78 | 10.61 |
|  | 24 | 3.54 | 2.78 | 4.72 | 3.63 | 5.57 | 4.3 | 5.01 | 4.95 | 5.51 | 5.31 | 7.51 | 6.92 | 6.1 | 5.1 | 6.4 | 6.4 | 7.68 | 7.4 | 10.41 | 8.44 | 11 | 8.8 |
|  | 26 | 2.86 | 2.23 | 3.74 | 2.97 | 5.6 | 4.29 | 6.15 | 5.22 | 6.99 | 6 | 7.33 | 6.5 | 7.9 | 6.1 | 8 | 7.08 | 8.8 | 8.4 | 10.56 | 9.46 | 10.6 | 9.8 |
|  | 27 | 2.54 | 2.16 | 3.85 | 3.08 | 6.8 | 4.54 | 6.08 | 5.62 | 7.37 | 4.51 | 8.9 | 5.27 | 8.69 | 6.5 | 9.27 | 7.04 | 11.7 | 7.85 | 10.66 | 8.2 | 13.44 | 8.86 |
| TRMBE | 32 | 4.31 | 3.4 | 5.35 | 4.53 | 5.54 | 4.05 | 7.01 | 5.4 | 7.38 | 6.97 | 8.46 | 8.27 | 9.07 | 6.92 | 8.7 | 8.7 | 9.56 | 8.52 | 10.55 | 10.33 | 13.58 | 13.03 |
|  | 35 | 4.78 | 2.85 | 4.79 | 3.17 | 4.59 | 3.26 | 7.68 | 5.5 | 9.74 | 6.95 | 11.13 | 6.58 | 12.8 | 6.94 | 12.56 | 6.95 | 14.8 | 8.32 | 15.8 | 10.7 | 16.5 | 11.7 |
|  | 36 | 4.23 | 2.95 | 4.22 | 3.77 | 6.07 | 4.43 | 6.8 | 5.29 | 6.7 | 5.88 | 8.67 | 7.33 | 9.2 | 7.65 | 9.37 | 9.1 | 12 | 11.38 | 14.3 | 11.7 | 15.8 | 13 |
|  | 40 | 4.02 | 2.48 | 5.08 | 4.13 | 6.02 | 5.56 | 8.16 | 4.96 | 8.47 | 5.75 | 10.8 | 6.1 | 10 | 7.84 | 12.72 | 8.14 | 12.93 | 9.75 | 14 | 9.2 | 14.69 | 10.37 |
|  | 41 | 4.01 | 2.27 | 4.93 | 3.96 | 4.3 | 4.3 | 5.91 | 5.26 | 6.41 | 5.49 | 8.47 | 7.42 | 9 | 6.76 | 11.7 | 8 | 12.95 | 10.15 | 13.4 | 12 | 13 | 12.37 |
|  | 42 | 4.24 | 1.38 | 5.15 | 3.91 | 5.87 | 4.33 | 8.73 | 6.89 | 7.92 | 7.26 | 8.34 | 7.8 | 7.6 | 6.8 | 10 | 8.7 | 12 | 10.67 | 13.12 | 12 | 15.5 | 13.16 |
| Combine | 46 | 4.76 | 3.54 | 4.65 | 4.12 | 4.92 | 3.62 | 6.52 | 4 | 6.06 | 3.97 | 6.74 | 4.77 | 7.3 | 5.4 | 8.3 | 6 | 12.07 | 7.53 | 11.41 | 7.49 | 11.8 | 8.1 |
|  | 49 | 4.3 | 3.16 | 3.72 | 3.16 | 5.67 | 3.48 | 5.66 | 5.59 | 8.58 | 4.99 | 8.96 | 5.14 | 9.87 | 6.66 | 11.58 | 5.98 | 13.26 | 6.28 | 13.33 | 7.1 | 13.51 | 7.27 |
|  | 51 | 3.99 | 2.92 | 5.31 | 4.07 | 5.26 | 3.24 | 5.27 | 4.27 | 4.51 | 3.99 | 5.56 | 3.52 | 6.3 | 5.78 | 7.24 | 5.2 | 7.7 | 5.74 | 8.58 | 7.06 | 9.3 | 7.8 |
|  | 53 | 3.46 | 2.9 | 4.13 | 3.56 | 3.88 | 2.96 | 5.2 | 3.64 | 5.62 | 4.04 | 5.5 | 3.67 | 5.8 | 4.8 | 6.8 | 5.6 | 7.8 | 5.4 | 8.15 | 6.31 | 8.45 | 6.26 |
|  | 55 | 3.79 | 2.44 | 3.81 | 2.88 | 4.8 | 3.01 | 6.5 | 4.85 | 4.1 | 3.47 | 5.38 | 4.4 | 6.18 | 5.49 | 7.77 | 7.23 | 9.86 | 6.94 | 9.8 | 7.03 | 9.91 | 7.5 |
|  | 56 | 3.85 | 2.28 | 4.95 | 4.05 | 4.52 | 4.32 | 6.05 | 4.84 | 5.97 | 5.16 | 7.33 | 5.43 | 6.9 | 5.6 | 8.24 | 6.95 | 9.47 | 6.94 | 9.51 | 7.6 | 10.17 | 8.8 |

LD: long diameter

SD: short diameter

**The tumor weights (TW) of subcutaneous GC mice in four therapy groups (g).**

| Vehicle | | TRMBE | | 5-FU | | Combine | |
| --- | --- | --- | --- | --- | --- | --- | --- |
| No. | TW | No. | TW | No. | TW | No. | TW |
| 1 | 1.3646 | 32 | 1.0099 | 16 | 0.3732 | 46 | 0.2883 |
| 2 | 1.5584 | 35 | 0.7499 | 18 | 0.3933 | 49 | 0.1921 |
| 7 | 0.6785 | 36 | 1.3195 | 22 | 0.334 | 51 | 0.1242 |
| 8 | 0.8197 | 40 | 0.8667 | 24 | 0.3143 | 53 | 0.1191 |
| 10 | 0.6193 | 41 | 0.5232 | 26 | 0.4325 | 55 | 0.1114 |
| 12 | 1.2131 | 42 | 1.1866 | 27 | 0.3675 | 56 | 0.2274 |

**The original tumor images of subcutaneous GC mice in four therapy groups.**

Vehicle	5-FU	TRMBE	Combine

Combine

**The original HE images (10x) of subcutaneous GC mice in four therapy groups.**

|  | Vehicle | 5-FU | TRMBE | Combine |
| --- | --- | --- | --- | --- |
| Lung |  |  |  |  |
| Liver |  |  |  |  |
| Brain |  |  |  |  |
| Spleen |  |  |  |  |
| Kidney |  |  |  |  |
| Heart |  |  |  |  |
| Tumor |  |  |  |  |

**The body weights (BW) of subcutaneous GC mice in four therapy groups (g).**

| Group | No. | Day 0 | Day 1 | Day 2 | Day 3 | Day 4 | Day 5 | Day 6 | Day 7 | Day 8 | Day 9 | Day 10 | Day 11 | Day 12 | Day 13 |
| --- | --- | --- | --- | --- | --- | --- | --- | --- | --- | --- | --- | --- | --- | --- | --- |
| Vehicle | 1 | 25.2 | 25.4 | 26.1 | 26.2 | 25.8 | 26.9 | 26.6 | 26.6 | 27.1 | 27.9 | 27.6 | 27 | 27 | 26.7 |
|  | 2 | 22.4 | 22.2 | 23 | 23.7 | 23.2 | 24.5 | 23.8 | 23.9 | 24.1 | 25.2 | 25.8 | 25.5 | 25.1 | 24.7 |
|  | 7 | 24.8 | 24.6 | 24.5 | 21.6 | 24 | 25.4 | 25.7 | 26 | 25.2 | 25.9 | 26 | 25.6 | 25.3 | 25.6 |
|  | 8 | 21.3 | 21.6 | 21.9 | 22.2 | 23.2 | 23.5 | 23.9 | 23.5 | 23.6 | 24.6 | 25.2 | 24.7 | 25.6 | 25.1 |
|  | 10 | 22.5 | 23.5 | 23.6 | 24.4 | 24.3 | 25.2 | 25 | 25.7 | 25.1 | 25.1 | 25.4 | 24.7 | 24.6 | 24.9 |
|  | 12 | 22.3 | 22.6 | 22.7 | 22.9 | 22.1 | 23.1 | 23.5 | 23.5 | 23.7 | 24.4 | 24.9 | 24.7 | 25.2 | 25.7 |
| 5-FU | 16 | 23.3 | 23.7 | 23.8 | 24 | 22.7 | 24.5 | 22.4 | 24.7 | 23.6 | 24.6 | 24.7 | 24.7 | 25.1 | 24.8 |
|  | 18 | 22 | 22.1 | 22.1 | 22.7 | 22.4 | 22.5 | 22.1 | 22.8 | 21.6 | 22.5 | 22.3 | 22.3 | 23.3 | 23.2 |
|  | 22 | 25.6 | 25.1 | 25.4 | 26 | 24.3 | 25.8 | 23.7 | 24.9 | 26.7 | 26.5 | 27.2 | 26.3 | 27.3 | 26.8 |
|  | 24 | 22.8 | 22.9 | 22.2 | 22.8 | 22.3 | 22.9 | 22 | 22 | 21.6 | 21.5 | 22.3 | 21 | 22.8 | 22.5 |
|  | 26 | 24.7 | 24.7 | 24.2 | 24.9 | 25.1 | 25 | 24.7 | 24.9 | 24.3 | 24.8 | 25.3 | 24.6 | 25 | 24.6 |
|  | 27 | 21.4 | 22 | 21.9 | 22.3 | 21.7 | 22.2 | 20 | 21.4 | 21.7 | 21.8 | 21.7 | 21.3 | 23.1 | 23.6 |
| TRMBE | 32 | 24.9 | 25.1 | 24.8 | 24.4 | 24.9 | 26.1 | 25 | 25.2 | 24.5 | 25.8 | 25.8 | 25.6 | 25.5 | 25.6 |
|  | 35 | 20 | 20 | 19.6 | 20.3 | 19 | 20.9 | 20 | 20.7 | 20.1 | 21.3 | 21.6 | 21.7 | 21.2 | 20.8 |
|  | 36 | 20.9 | 20.7 | 20.9 | 21.9 | 22.1 | 23.3 | 21.9 | 22.1 | 21.2 | 23 | 24.1 | 24 | 24.1 | 24.4 |
|  | 40 | 25.5 | 24.6 | 24.6 | 24.5 | 24.8 | 26.1 | 24.5 | 25 | 24.9 | 26.4 | 27.6 | 27.3 | 26.7 | 26.6 |
|  | 41 | 21.3 | 21.6 | 21.1 | 21.1 | 20.9 | 22 | 22.1 | 21.5 | 20.7 | 22.2 | 22.4 | 22 | 22.5 | 21.4 |
|  | 42 | 22 | 20.7 | 21.2 | 21.4 | 21.4 | 22 | 21.8 | 21.6 | 21.2 | 22 | 22.2 | 21.8 | 21.9 | 21 |
| Combine | 46 | 20.4 | 21.4 | 22.1 | 21.9 | 21.3 | 22.5 | 23.5 | 22.8 | 21.9 | 21.9 | 21.8 | 22.3 | 23.8 | 24 |
|  | 49 | 21.3 | 21.8 | 21.4 | 22.1 | 22.5 | 23.3 | 22.9 | 24.6 | 23.3 | 25.3 | 25.1 | 24.5 | 24.7 | 23.6 |
|  | 51 | 21.4 | 22.1 | 21.5 | 20.9 | 21.4 | 22.3 | 21.3 | 22.4 | 22.1 | 23.2 | 22.9 | 22.1 | 25.3 | 24.5 |
|  | 53 | 22.6 | 22.2 | 22 | 21.7 | 22.2 | 21.4 | 22.3 | 23.7 | 23.7 | 25.4 | 23.8 | 23.8 | 25.4 | 24 |
|  | 55 | 23.7 | 23.9 | 23.9 | 23.4 | 22.8 | 23.5 | 23.9 | 25.4 | 25 | 26 | 25.6 | 24.2 | 26.6 | 26.8 |
|  | 56 | 22 | 21.7 | 21.2 | 21.7 | 21.6 | 21.8 | 22.6 | 26.9 | 22.9 | 23.9 | 24.1 | 23.6 | 25.8 | 25.6 |

1.4 **The data of survival mice model.**

| Vehicle | | | 5-FU | | | TRMBE | | | Combine | | |
| --- | --- | --- | --- | --- | --- | --- | --- | --- | --- | --- | --- |
| No. | status (1=died; 0= live) | times (day) | No. | status (1=died; 0= live) | times (day) | No. | status (1=died; 0= live) | times (day) | No. | status (1=died; 0= live) | times (day) |
| 1 | 1 | 13 | 11 | 1 | 16 | 21 | 1 | 12 | 31 | 1 | 18 |
| 2 | 1 | 12 | 12 | 1 | 16 | 22 | 1 | 13 | 32 | 1 | 18 |
| 3 | 1 | 14 | 13 | 1 | 15 | 23 | 1 | 13 | 33 | 1 | 17 |
| 4 | 1 | 12 | 14 | 1 | 16 | 24 | 1 | 12 | 34 | 1 | 16 |
| 5 | 1 | 12 | 15 | 1 | 16 | 25 | 1 | 12 | 35 | 1 | 19 |
| 6 | 1 | 12 | 16 | 1 | 16 | 26 | 1 | 13 | 36 | 1 | 17 |
| 7 | 1 | 12 | 17 | 1 | 18 | 27 | 1 | 12 | 37 | 1 | 17 |
| 8 | 1 | 13 | 18 | 1 | 15 | 28 | 1 | 15 | 38 | 1 | 18 |
| 9 | 1 | 11 | 19 | 1 | 15 | 29 | 1 | 13 | 39 | 0 | 22 |
| 10 | 1 | 12 | 20 | 1 | 16 | 30 | 1 | 12 | 40 | 1 | 18 |

1.5 **The data of** **liver metastasis model.**

**The body weights of liver metastasis mice in four therapy groups (g).**

|  | No. | Day 0 | Day 2 | Day 4 | Day 6 | Day 8 | Day 10 | Day 12 | Day 14 |
| --- | --- | --- | --- | --- | --- | --- | --- | --- | --- |
| Vehicle | 28 | 25.3 | 26.5 | 26.5 | 27.1 | 27.1 | 28.2 | 27.5 | 27.9 |
|  | 16 | 21.3 | 23.2 | 22.2 | 23 | 22.5 | 22.7 | 22.7 | 22.2 |
|  | 36 | 25.2 | 25.8 | 25.6 | 26.7 | 26.1 | 26.3 | 26.4 | 26.8 |
|  | 10 | 24.8 | 25.3 | 24.7 | 25 | 24.7 | 24.9 | 24.9 | 24.8 |
|  | 25 | 20.6 | 20.9 | 21 | 20.4 | 20.3 | 21.5 | 21.4 | 21.2 |
|  | 26 | 26 | 26.3 | 27.3 | 26.5 | 27.6 | 28.7 | 28.1 | 28 |
| TRMBE | 35 | 25.1 | 25.5 | 24.9 | 25 | 25.8 | 26.4 | 25.5 | 24.8 |
|  | 14 | 23 | 22.8 | 23.2 | 22.8 | 23.5 | 24.3 | 23.9 | 23.8 |
|  | 11 | 25.5 | 26.4 | 26.3 | 25.3 | 25.8 | 27 | 26.3 | 25.8 |
|  | 9 | 24.9 | 25.2 | 25.4 | 25.2 | 24.9 | 26.4 | 25.8 | 25 |
|  | 18 | 23.9 | 24.2 | 23.2 | 23.8 | 23.3 | 24.1 | 23.9 | 24.1 |
|  | 41 | 30.4 | 31 | 30.4 | 31.3 | 30.9 | 32.5 | 32.5 | 33.3 |
| 5-FU | 15 | 26 | 27 | 27.6 | 26.5 | 24.9 | 25.5 | 26.1 | 25.5 |
|  | 2 | 23.7 | 23.8 | 23.3 | 23.2 | 23.7 | 24 | 24.4 | 23.7 |
|  | 6 | 25.4 | 25.8 | 25.7 | 24.3 | 22.6 | 23.1 | 24.3 | 24.4 |
|  | 4 | 23.6 | 24.6 | 23.6 | 23.2 | 23.2 | 24.9 | 24.9 | 23.7 |
|  | 17 | 26.7 | 27.7 | 25.5 | 24.5 | 22.7 | 24 | 24.6 | 24.4 |
|  | 29 | 24.1 | 24.6 | 23.1 | 22 | 21.2 | 21.5 | 22.9 | 22.8 |
| Combine | 24 | 23.9 | 25 | 23.7 | 23 | 20.8 | 20.9 | 19.1 | 17.6 |
|  | 20 | 25.5 | 26.3 | 25.9 | 25.7 | 24.8 | 25.1 | 26 | 25.4 |
|  | 12 | 26.4 | 26.5 | 26.4 | 24.8 | 24.5 | 25 | 23.9 | 24.8 |
|  | 37 | 26.3 | 27.3 | 26.3 | 25.8 | 24.6 | 26.8 | 26.1 | 26 |
|  | 23 | 24.8 | 25 | 24.6 | 24.5 | 23.6 | 25 | 24.8 | 24.7 |
|  | 22 | 25.8 | 26 | 25.5 | 24.1 | 22 | 22.8 | 23.3 | 23.2 |

**The Liver weights (LW) of liver metastasis mice in four therapy groups (g).**

| Vehicle | | TRMBE | | 5-FU | | Combine | |
| --- | --- | --- | --- | --- | --- | --- | --- |
| No. | LW | No. | LW | No. | LW | No. | LW |
| 28 | 1.986 | 35 | 2.447 | 15 | 1.778 | 24 | 1.4596 |
| 16 | 1.9016 | 14 | 1.769 | 2 | 1.7377 | 20 | 1.415 |
| 36 | 1.547 | 11 | 1.688 | 6 | 1.735 | 12 | 1.2815 |
| 10 | 1.473 | 9 | 1.604 | 4 | 1.5834 | 37 | 1.1477 |
| 25 | 1.398 | 18 | 1.43 | 17 | 1.404 | 23 | 0.9564 |
| 26 | 1.371 | 41 | 1.4 | 29 | 1.435 | 22 | 0.909 |

**The numbers of surface liver metastasis**

| 5-FU | | Combine | |
| --- | --- | --- | --- |
| No. | numbers | No. | numbers |
| 15 | 98 | 24 | 31 |
| 2 | 46 | 20 | 23 |
| 6 | 86 | 12 | 35 |
| 4 | 266 | 37 | 5 |
| 17 | 76 | 23 | 9 |
| 29 | 86 | 22 | 23 |

**The original liver metastasis tumor in four therapy groups (g).**

Vehicle	TRMBE

5-FU	Combine

**The original data of ALT and AST in liver metastasis tumor.**

|  | Vehicle | TRMBE | 5-FU | Combine |
| --- | --- | --- | --- | --- |
| **ALT (U/L)** | 551.3 | 433.5 | 131.7 | 74.4 |
|  | 604.1 | 526.7 | 120.2 | 68 |
|  | 463.5 | 577.2 | 131.5 | 59.7 |
| **AST (U/L)** | 646.6 | 525.6 | 240.1 | 194.5 |
|  | 800.8 | 617.5 | 280.3 | 161.8 |
|  | 589.2 | 692.4 | 305.5 | 143.7 |

1.6 **The original data of cytokines in peripheral blood (subcutaneous GC model)**

| (pg/ml) | 1 | 2 | 7 | 36 | 32 | 35 | 16 | 18 | 22 | 46 | 51 | 49 |
| --- | --- | --- | --- | --- | --- | --- | --- | --- | --- | --- | --- | --- |
| IL-6 | 55.8 | 49.7 | 54.5 | 69 | 58.4 | 53.3 | 47.8 | 39.9 | 52.9 | 12.9 | 15.7 | 16.8 |
| IL-10 | 195 | 209.2 | 153.8 | 131.9 | 146.1 | 176.6 | 168.1 | 121 | 170.7 | 48.1 | 63.6 | 43.2 |
| IFNg | 21.8 | 20.6 | 13 | 10.6 | 20 | 20 | 16.7 | 25.8 | 27.1 | 41.1 | 48 | 49.5 |
| TGFb1 | 963.7 | 1,040.90 | 1,287.60 | 569.7 | 693.2 | 531 | 496.3 | 635.2 | 624.5 | 214 | 172.2 | 193 |
| IL-1b | 11 | 8 | 5.6 | 8.2 | 10.9 | 6.5 | 10.7 | 17.9 | 11.7 | 3.2 | 8.9 | 12.2 |
| IL-2 | 10.1 | 9.6 | 4.8 | 2.8 | 9.6 | 7.3 | 6.7 | 12.5 | 15.8 | 7 | 10.6 | 17.3 |
| IL-4 | 0.1 | 0.1 | 0.4 | 0.3 | 0.4 | 0.2 | 0.3 | 0.4 | 0.7 | 0.3 | 0.2 | 0.8 |
| IL-5 | 8.5 | 11.4 | 15.9 | 14.9 | 15.2 | 12.4 | 7 | 27 | 25.4 | 6.7 | 19 | 28.8 |
| IL-12p70 | 3.7 | 5 | 7.2 | 2.7 | 12 | 4.1 | 3.3 | 2.9 | 8.1 | 10.6 | 7.8 | 6.2 |
| IL-13 | 10.6 | 0.1 | 4.2 | 1.6 | 8.7 | 12.5 | 2.8 | 10.8 | 14.3 | 5.7 | 7.5 | 15.5 |
| IL-17 | 20.9 | 2.6 | 8 | 2.7 | 5.6 | 19.1 | 5.4 | 14.2 | 5.4 | 13.5 | 3.3 | 12 |
| IL-17F | 20.5 | 11.4 | 14.8 | 19.2 | 11.2 | 11.6 | 10.5 | 14.5 | 13.7 | 21.2 | 13.1 | 15.4 |
| IL-21 | 1.1 | 6.1 | 3.4 | 5.5 | 6 | 1.5 | 6.7 | 1 | 4.1 | 11 | 0.9 | 5 |
| IL-22 | 12.7 | 0 | 0 | 11.7 | 0 | 0.9 | 0 | 11 | 0.7 | 4 | 0 | 6.2 |
| IL-23 | 175.7 | 48.2 | 128.3 | 170.7 | 118.9 | 71.3 | 111.1 | 161.3 | 176.6 | 184.6 | 122.7 | 217.6 |
| IL-28 | 2.9 | 4.7 | 12.7 | 7.3 | 5.4 | 7.6 | 2.8 | 5.6 | 17.1 | 17.1 | 6.5 | 10.6 |
| MIP-3a | 35.6 | 39.9 | 93 | 74.2 | 28 | 95.5 | 47.3 | 125.1 | 38.4 | 129.1 | 51.6 | 129.5 |
| IL-6 | 55.8 | 49.7 | 54.5 | 69 | 58.4 | 53.3 | 47.8 | 39.9 | 52.9 | 12.9 | 15.7 | 16.8 |

1.7 **The original data of immune cells in spleen (subcutaneous GC model)**

|  | Vehicle | | | TRMBE | | | 5-FU | | | Combine | | |
| --- | --- | --- | --- | --- | --- | --- | --- | --- | --- | --- | --- | --- |
| NO. | 2 | 1 | 7 | 35 | 32 | 36 | 16 | 18 | 22 | 51 | 53 | 56 |
| CD3/CD45 | 4.91 % | 9.23 % | 8.93 % | 12.3 % | 12.1 % | 12.4 % | 12.9 % | 13.1 % | 12.6 % | 18.2 % | 14.1 % | 14.2 % |
| CD4/CD45 | 2.86% | 5.49 % | 6.16 % | 8.16 % | 8.15 % | 8.42 % | 8.89 % | 8.24 % | 8.28 % | 10.6 % | 9.42 % | 9.44 % |
| CD8/CD45 | 1.31% | 2.70 % | 2.26 % | 3.31 % | 3.21 % | 3.19 % | 3.16 % | 3.79 % | 3.39 % | 6.51 % | 3.93 % | 3.92 % |
| Treg/CD45 | 1.15 % | 0.67 % | 0.51 % | 1.28 % | 1.37 % | 1.39 % | 1.36 % | 1.54 % | 1.33 % | 2.09 % | 1.37 % | 1.35 % |
| B/CD45 | 67.1 % | 55.4 % | 67.0 % | 76.2 % | 77.0 % | 76.2 % | 70.0 % | 71.0 % | 76.4 % | 73.0 % | 77.7 % | 76.6 % |
| NK/CD45 | 1.90 % | 0.89 % | 0.74 % | 0.68 % | 0.68 % | 0.73 % | 0.50 % | 0.99 % | 0.72 % | 0.50 % | 0.51 % | 0.54 % |
| DC/CD45 | 1.72 % | 2.39 % | 3.71 % | 1.61 % | 1.06 % | 1.18 % | 2.04 % | 2.63 % | 1.73 % | 1.07 % | 0.56 % | 0.68 % |
| M/CD45 | 10.0 % | 12.6 % | 6.77 % | 2.12 % | 1.92 % | 1.76 % | 4.71 % | 3.30 % | 1.91 % | 0.87 % | 1.49 % | 1.70 % |
| PMN-MDSCs/CD45 | 4.98 % | 4.90 % | 3.95 % | 0.46 % | 0.30 % | 0.28 % | 1.29 % | 1.65 % | 0.48 % | 0.026 % | 0.17 % | 0.15 % |
| M-MDSCs/CD45 | 1% | 0.54 % | 0.33 % | 0.088 % | 0.028 % | 0.018 % | 2.00 % | 1.52 % | 0.071 % | 0.060 % | 0.037 % | 0.036 % |

1.8 **The original data of immune cells in TME (subcutaneous GC model)**

|  | Vehicle | | | TRMBE | | | 5-FU | | | Combine | | |
| --- | --- | --- | --- | --- | --- | --- | --- | --- | --- | --- | --- | --- |
| NO. | 1 | 2 | 7 | 35 | 36 | 32 | 16 | 22 | 18 | 51 | 56 | 53 |
| CD3/CD45 | 4.27% | 4.48% | 4.59% | 2.36% | 9.03% | 3.22% | 2.31% | 3.36% | 2.45% | 8.21% | 12.90% | 17.40% |
| CD8/CD45 | 1.57 % | 1.13 % | 1.29 % | 0.87 % | 2.37 % | 0.71 % | 0.69 % | 1.19 % | 1.07 % | 1% | 1.42 % | 2.58 % |
| PD1/CD8 | 90.5% | 87.1% | 83.2% | 81.7% | 83.8% | 88.7% | 78.5% | 87.7 % | 84.2% | 71.4% | 68.7% | 63.8% |
| CD4/CD45 | 1.16 % | 1.88 % | 1.26 % | 1.05 % | 3.52 % | 1.49 % | 1.11 % | 0.84 % | 0.65 % | 2.70 % | 8.46 % | 9.16 % |
| TREG/CD45 | 0.75 % | 0.86 % | 0.95 % | 0.58 % | 2.56 % | 1.13 % | 0.53 % | 0.56 % | 0.44 % | 3% | 4.98 % | 4.18 % |
| NK/CD45 | 1.47 % | 2.32 % | 1.10 % | 1.19 % | 1.55 % | 0.84 % | 1% | 1.25 % | 1.21 % | 4% | 4.96 % | 6.01 % |
| DC/CD45 | 14.6 % | 12.6 % | 10.3 % | 16.6 % | 7.99 % | 11.5 % | 15.4 % | 13.1 % | 14.4 % | 8.23 % | 7.27 % | 9.90 % |
| Mø/CD45 | 4.06 % | 4.68 % | 4.37 % | 9.39 % | 7.68 % | 11.8 % | 8.01 % | 6.20 % | 9.20 % | 9.63 % | 15.8 % | 15.8 % |
| PMN-MDSCs/CD45 | 42.0 % | 40.0 % | 40.5 % | 21.9 % | 26.7 % | 29.7 % | 37.9 % | 35.8 % | 34.5 % | 10.5 % | 11.9 % | 8.76 % |
| M-MDSCs/CD45 | 6.82 % | 10.2 % | 5.95 % | 3.86 % | 11.9 % | 11.7 % | 6.94 % | 6.79 % | 6.76 % | 1.83 % | 1.36 % | 2.70 % |

1.9 **The original data of** s**pecific immune cell depletion model.**

**The original data of CD8+T and NK cells in blood**

| Percent of CD8+ T/CD45+ cell | | | | | |
| --- | --- | --- | --- | --- | --- |
| Combine | | Combine+CD8^+^T depletion | | Combine+NK depletion | |
| 13.60% | | 0% | | 20.20% | |
| 12.90% | | 0.00% | | 20.20% | |
| 19.70% | | 0.02% | | 16.60% | |
| Percent of NK/CD45+ cell | | |  | |  |
| Combine | Combine+CD8^+^T depletion | | Combine+NK depletion | |  |
| 4.75 | 4.34 | | 0.15 | |  |
| 5.29 | 4.48 | | 0.2 | |  |
| 6 | 6.36 | | 0.047 | |  |

**The tumor long and short diameters of subcutaneous GC mice in five therapy groups (mm).**

| Group | No. | Day 3 | | Day 4 | | Day 5 | | Day 6 | | Day 7 | | Day 8 | | Day 9 | | Day 10 | | Day 11 | | Day 12 | | Day 13 | |
| --- | --- | --- | --- | --- | --- | --- | --- | --- | --- | --- | --- | --- | --- | --- | --- | --- | --- | --- | --- | --- | --- | --- | --- |
|  |  | LD | SD | LD | SD | LD | SD | LD | SD | LD | SD | LD | SD | LD | SD | LD | SD | LD | SD | LD | SD | LD | SD |
| Vehicle | 12 | 6.14 | 5.25 | 6.32 | 5.85 | 8.26 | 4.57 | 6.74 | 5.72 | 8.32 | 7.85 | 10.61 | 9.39 | 11.16 | 9.49 | 13.01 | 10.2 | 13.99 | 10.45 | 13.79 | 13.16 | 15.51 | 14.75 |
|  | 49 | 5.1 | 4.56 | 5.93 | 5.01 | 6.33 | 5.67 | 7.86 | 6.44 | 7.93 | 7.01 | 10.79 | 8 | 11.02 | 8.65 | 11.4 | 8.73 | 16.93 | 9.46 | 18.5 | 10.76 | 15.32 | 12.75 |
|  | 6 | 5.4 | 4 | 7.64 | 5.67 | 9.49 | 6.14 | 8.75 | 7.04 | 9.64 | 7.67 | 9.03 | 7.4 | 9.39 | 8.25 | 11.97 | 9.67 | 13.14 | 10.59 | 12.73 | 11.65 | 14.4 | 12.78 |
|  | 27 | 4.39 | 4.22 | 6.11 | 4.33 | 7.62 | 5.1 | 7.01 | 6.81 | 8.11 | 6.33 | 9.73 | 7.31 | 9.81 | 8.3 | 12.17 | 9.42 | 11.65 | 10.31 | 12.55 | 11.89 | 15.43 | 12.85 |
|  | 5 | 4.06 | 3.3 | 4.7 | 4.69 | 6.38 | 5.49 | 6.99 | 5.48 | 6.7 | 6.69 | 9.59 | 7.31 | 10.02 | 7.51 | 10.34 | 8 | 11.39 | 9.17 | 12.47 | 11.79 | 14.3 | 13.2 |
|  | 28 | 3.01 | 2.95 | 5.06 | 3.86 | 6.07 | 3.97 | 7.55 | 5.02 | 7.06 | 5.86 | 9.9 | 6.85 | 9.95 | 7.93 | 10.7 | 8.01 | 12.31 | 9.3 | 13.09 | 11.74 | 14.76 | 11.94 |
| 5-FU | 38 | 5.35 | 5.18 | 5.48 | 5.3 | 7.16 | 5.61 | 6.38 | 5.48 | 6.98 | 6.8 | 9.4 | 7.41 | 8.88 | 7.57 | 9.16 | 8.99 | 10.29 | 9 | 10.76 | 10.67 | 11.6 | 11.6 |
|  | 24 | 4.92 | 4.32 | 5.84 | 5.1 | 6.97 | 5.25 | 6.01 | 5.52 | 6.34 | 6.1 | 6.94 | 6.69 | 7.45 | 7.01 | 8.05 | 7.96 | 8.83 | 8.47 | 11.35 | 10.15 | 12.47 | 11.61 |
|  | 8 | 4.5 | 4.21 | 6.12 | 5.09 | 6.81 | 5.48 | 7.22 | 5.6 | 6.62 | 6.59 | 7.12 | 6.87 | 7.43 | 6.54 | 7.81 | 7.48 | 10.15 | 9.45 | 11.29 | 11.23 | 11.18 | 11.71 |
|  | 22 | 5.21 | 3.82 | 6.25 | 5 | 7.47 | 5.9 | 7.03 | 6.39 | 7.75 | 6.5 | 8.26 | 7.14 | 8.57 | 8.05 | 9.49 | 8.21 | 10.92 | 9.9 | 10.48 | 10.22 | 12.98 | 11.49 |
|  | 40 | 4.17 | 3.2 | 7.16 | 4.22 | 8.8 | 5.09 | 8.95 | 5.52 | 9.13 | 5.72 | 12.84 | 6.07 | 11.75 | 6.3 | 11.96 | 7.79 | 14.96 | 7.25 | 12.48 | 9.33 | 13.15 | 9.93 |
|  | 30 | 3.12 | 3.05 | 5.76 | 4.48 | 6.6 | 5.46 | 6.47 | 4.86 | 7.26 | 5.98 | 8.11 | 5.74 | 8.54 | 6.19 | 8.84 | 8.41 | 11.3 | 8.45 | 11.55 | 7.66 | 12.33 | 11.09 |
| Combine | 44 | 5.15 | 4.6 | 5.16 | 4.74 | 5.05 | 4.53 | 6.1 | 5.31 | 6.16 | 5.36 | 7.05 | 5.13 | 6.87 | 5.15 | 7.13 | 5.71 | 12.86 | 6.45 | 12.15 | 7.18 | 12.42 | 8.47 |
|  | 41 | 5.33 | 4.27 | 4.8 | 4.8 | 4.55 | 4.35 | 5.05 | 4.38 | 5.8 | 4.8 | 5.93 | 5.17 | 6.29 | 5.07 | 6 | 4.9 | 7.44 | 6.7 | 8.2 | 6.84 | 8.52 | 7.96 |
|  | 20 | 5.26 | 4.16 | 5.39 | 4.45 | 5.91 | 4.61 | 7.16 | 4.49 | 6.39 | 5.45 | 6.89 | 4.58 | 7.08 | 5.23 | 7.54 | 5.5 | 8.8 | 7.11 | 9.04 | 8.83 | 9.76 | 8.93 |
|  | 9 | 4.37 | 4.24 | 5.79 | 5.2 | 7.43 | 5.16 | 5.67 | 4.92 | 6.79 | 5.2 | 6.56 | 4.92 | 7.77 | 6.16 | 7.8 | 6.41 | 7.31 | 6.97 | 8 | 7.59 | 8.86 | 7.64 |
|  | 50 | 4.81 | 3.91 | 4.6 | 4.32 | 5.71 | 5.62 | 8.11 | 5.25 | 8.6 | 5.32 | 11.27 | 6.01 | 11.08 | 5.6 | 12.08 | 5.74 | 14.36 | 5.84 | 12.32 | 6.4 | 12.32 | 6.4 |
|  | 26 | 4.36 | 4 | 4.98 | 4.58 | 5.2 | 4.93 | 5.29 | 4.04 | 5.98 | 5.58 | 7.96 | 6.8 | 7.76 | 6.71 | 7.72 | 6.41 | 8.65 | 6.53 | 9.79 | 6.84 | 9.54 | 9.13 |
| Combine + CD8+T depletion | 37 | 7.2 | 4.6 | 6.79 | 4.47 | 6.16 | 5.1 | 7.46 | 5.9 | 7.79 | 5.47 | 7.01 | 6.35 | 7.81 | 7.55 | 9.85 | 8.88 | 10.38 | 9.23 | 10.57 | 10.05 | 13.23 | 9.67 |
|  | 10 | 6.51 | 4.58 | 7.15 | 5.08 | 8.13 | 5.2 | 8.28 | 5.84 | 8.15 | 6.08 | 8.5 | 6.72 | 9.46 | 6.97 | 10.48 | 6.67 | 12.71 | 9.1 | 12.29 | 8.65 | 13.45 | 9.67 |
|  | 29 | 4.91 | 4.53 | 5.5 | 4.51 | 5.01 | 4.8 | 7.42 | 6.15 | 6.5 | 6.34 | 7.8 | 7.12 | 8.42 | 8.3 | 8.99 | 8.77 | 10.33 | 9.22 | 10.88 | 9.18 | 12.34 | 11.48 |
|  | 42 | 4.74 | 4.23 | 5.66 | 5.4 | 6.33 | 6.05 | 8.06 | 5.92 | 6.66 | 6.4 | 8.07 | 7.24 | 8.23 | 7.46 | 10.33 | 7.06 | 11.01 | 9.37 | 11.83 | 9.26 | 12.23 | 11.26 |
|  | 35 | 4.17 | 4.14 | 6.7 | 5.26 | 7.56 | 5.86 | 6.78 | 5.28 | 8.21 | 6.03 | 8.46 | 7.55 | 8.13 | 7.35 | 8.98 | 7.92 | 9.33 | 7.53 | 11.2 | 10.19 | 11.95 | 10.47 |
|  | 3 | 3.43 | 3.35 | 5.4 | 4.92 | 6.51 | 6.35 | 7.65 | 5.27 | 7.66 | 6.92 | 7.7 | 7.4 | 7.81 | 7.59 | 9.44 | 7.41 | 10.13 | 7.12 | 10.28 | 9.68 | 11.09 | 10.42 |
| Combine + NK depletion | 51 | 6.4 | 4.77 | 6.33 | 5.13 | 6.82 | 5.44 | 7.2 | 5.35 | 7.33 | 6.13 | 9.55 | 6.68 | 9.26 | 6.46 | 9.42 | 6.92 | 14.26 | 7.84 | 15.57 | 8.85 | 16.2 | 9.71 |
|  | 25 | 4.73 | 4.42 | 5.57 | 5.49 | 7.75 | 5.89 | 5.47 | 5.33 | 6.57 | 6.49 | 8.36 | 6.65 | 9.11 | 6.81 | 9.48 | 7.08 | 9.32 | 7.42 | 10.81 | 10 | 12.68 | 9.97 |
|  | 46 | 5.39 | 4.01 | 5.49 | 5.24 | 6.58 | 6.51 | 7.1 | 5.99 | 8.49 | 6.24 | 9.74 | 6.91 | 10.15 | 6.23 | 10.04 | 6.53 | 12.48 | 8.73 | 13.41 | 10 | 14.15 | 10.93 |
|  | 23 | 5.01 | 4.09 | 5.6 | 5.55 | 6.15 | 5.85 | 7.02 | 5.76 | 7.6 | 6.55 | 8.91 | 7.42 | 8.77 | 8.21 | 8.65 | 8.15 | 9.75 | 8.04 | 10.51 | 10.38 | 12.12 | 10.3 |
|  | 45 | 4.94 | 3.85 | 5.3 | 5.28 | 6.16 | 5.45 | 8.54 | 4.7 | 8.3 | 6.28 | 9.2 | 6.79 | 8.43 | 6.83 | 8.93 | 6.44 | 11.88 | 8.09 | 12.2 | 8.92 | 12.63 | 9.88 |
|  | 18 | 5.27 | 3.14 | 5.26 | 4.63 | 6.83 | 5.17 | 5.68 | 5.07 | 6.26 | 5.63 | 8.47 | 7.68 | 8.87 | 7.52 | 9.09 | 8.28 | 10.53 | 9.58 | 10.61 | 10.02 | 10.84 | 10.75 |

LD: long diameter

SD: short diameter

**The body weight of subcutaneous GC mice in five therapy groups (g).**

| Group | No. | DAY 0 | DAY 1 | DAY 2 | DAY 3 | DAY 4 | DAY 5 | DAY 6 | DAY 7 | DAY 8 | DAY 9 | DAY 10 | DAY 11 | DAY 12 | DAY 13 |
| --- | --- | --- | --- | --- | --- | --- | --- | --- | --- | --- | --- | --- | --- | --- | --- |
| Vehicle | 12 | 19.4 | 19.7 | 20.1 | 20.2 | 20.7 | 21.3 | 21.9 | 22.2 | 22.5 | 22.5 | 23.1 | 23.4 | 22.8 | 22 |
|  | 49 | 19.4 | 19.7 | 20.1 | 20.1 | 20.6 | 21.7 | 22.2 | 22 | 22.1 | 22.1 | 24 | 25.1 | 25.4 | 26.3 |
|  | 6 | 20.1 | 19.8 | 20.1 | 20.5 | 21 | 22 | 22.1 | 23.4 | 21.9 | 21.9 | 23.5 | 23.9 | 23.3 | 22.3 |
|  | 27 | 19.6 | 20.1 | 20 | 19.8 | 20.3 | 21.1 | 21.2 | 21.1 | 21.7 | 21.7 | 22.7 | 23.2 | 23.7 | 23.2 |
|  | 5 | 19.2 | 19.8 | 19.9 | 20.4 | 20.9 | 21.9 | 22.1 | 21.6 | 22.6 | 22.6 | 22.9 | 23.5 | 23.8 | 23.7 |
|  | 28 | 20.2 | 20.2 | 20 | 20.3 | 20.8 | 21.2 | 21.8 | 21.3 | 21.6 | 21.6 | 23.8 | 24.2 | 24.1 | 24.4 |
| 5-FU | 38 | 20.4 | 20.4 | 20.9 | 20.2 | 20.7 | 21.8 | 22.1 | 22.1 | 22.7 | 22.7 | 23.3 | 23.5 | 23.9 | 24.2 |
|  | 24 | 20.6 | 20.6 | 20.3 | 19.4 | 19.9 | 21.7 | 22 | 20.8 | 21.7 | 21.7 | 23 | 22.1 | 22.2 | 22.3 |
|  | 8 | 21.3 | 21.3 | 21.1 | 21.1 | 21.6 | 20.6 | 21.5 | 17.4 | 23 | 23 | 23.3 | 23.9 | 24.4 | 24.3 |
|  | 22 | 23.5 | 23.5 | 24.6 | 25 | 25.5 | 25.7 | 25.8 | 25.2 | 25.9 | 25.9 | 28 | 26.5 | 26.7 | 26.1 |
|  | 40 | 20.1 | 20.1 | 20.4 | 20.3 | 20.8 | 21.8 | 22.9 | 21.9 | 22.2 | 22.2 | 23 | 22.9 | 23.8 | 24.5 |
|  | 30 | 19.9 | 19.9 | 20.1 | 20.7 | 21.2 | 22.5 | 22.7 | 22.1 | 22.4 | 22.4 | 23.5 | 22.9 | 23.5 | 23.5 |
| Combine | 44 | 19.5 | 19.2 | 19.6 | 19.3 | 19.4 | 20.4 | 20.6 | 20.1 | 20.5 | 20.5 | 20.8 | 21.6 | 22.3 | 22.6 |
|  | 41 | 19.9 | 20.5 | 20.6 | 21.4 | 21.9 | 20.8 | 21.5 | 22.1 | 22.8 | 22.8 | 22.4 | 22.2 | 22.3 | 23.2 |
|  | 20 | 19.8 | 20.1 | 19.7 | 19.9 | 20.4 | 21 | 22.3 | 22.5 | 23.3 | 23.1 | 23.2 | 23.6 | 23.7 | 23.1 |
|  | 9 | 20.3 | 20.6 | 20.8 | 20.5 | 21 | 22 | 22.3 | 22 | 22.4 | 23.4 | 23.8 | 23.4 | 23.7 | 23.3 |
|  | 50 | 21.6 | 19.6 | 19.2 | 18.8 | 19.3 | 19.4 | 20.6 | 21.3 | 22 | 22.3 | 22.6 | 23.1 | 23.7 | 24.1 |
|  | 26 | 19.4 | 20.5 | 20.3 | 19.9 | 20.4 | 20.6 | 21.2 | 22.1 | 22.2 | 22.9 | 23.7 | 23.5 | 23.8 | 23.8 |
| Combine + CD8+T depletion | 37 | 19.8 | 20.8 | 20.8 | 20 | 20.5 | 21.8 | 22.8 | 22.2 | 21.5 | 21.5 | 22.5 | 22.2 | 23 | 24.1 |
|  | 10 | 20.4 | 21.3 | 21 | 21.7 | 22.2 | 23.5 | 23.6 | 23.3 | 23.2 | 23.2 | 25 | 25 | 25.3 | 25.4 |
|  | 29 | 19.8 | 20.2 | 20.3 | 19.5 | 20 | 21.6 | 22 | 21.3 | 21.4 | 21.4 | 22.4 | 22.2 | 22.6 | 22.6 |
|  | 42 | 20.6 | 21 | 22 | 22 | 22.5 | 23.2 | 24 | 23 | 23.5 | 23.5 | 24.6 | 24.4 | 24.9 | 25 |
|  | 35 | 20.1 | 20.6 | 21.2 | 20.9 | 21.4 | 22.4 | 23.5 | 22.5 | 23.5 | 23.5 | 24.2 | 24.1 | 24.6 | 24.1 |
|  | 3 | 19.7 | 20.1 | 21.4 | 21.7 | 22.2 | 22.5 | 23 | 21.8 | 22.9 | 22.9 | 24 | 24.7 | 24.3 | 24.1 |
| Combine + NK depletion | 51 | 20.6 | 21.8 | 21 | 21.2 | 21.7 | 22.2 | 22.8 | 22.9 | 23.1 | 23.1 | 23.8 | 23.9 | 24.7 | 25.1 |
|  | 25 | 19.9 | 20.3 | 19.9 | 19.2 | 19.7 | 20.7 | 21.6 | 20.9 | 21.2 | 21.2 | 22 | 22.3 | 22.8 | 23 |
|  | 46 | 20.6 | 20.9 | 19.9 | 20 | 20.5 | 20.6 | 21.4 | 21.1 | 21.3 | 21.3 | 22.7 | 22.9 | 23.3 | 23.4 |
|  | 23 | 20.8 | 21.1 | 20.1 | 19.3 | 19.8 | 20.7 | 20.9 | 20.3 | 20.5 | 20.5 | 22.1 | 21.7 | 23 | 22.5 |
|  | 45 | 19.7 | 20.4 | 19.4 | 19.1 | 19.6 | 20 | 21.1 | 20.1 | 20.6 | 20.6 | 21.4 | 21.9 | 23.1 | 22.2 |
|  | 18 | 19.2 | 19.5 | 19.6 | 18.8 | 19.3 | 20.3 | 21.4 | 20.8 | 20.9 | 20.9 | 22 | 21.8 | 21.4 | 21.9 |

**The tumor weight (TW) of subcutaneous GC mice in five therapy groups (g).**

| Vehicle | | 5-FU | | Combine | | Combine +CD8 depletion | | Combine +NK depletion | |
| --- | --- | --- | --- | --- | --- | --- | --- | --- | --- |
| No. | TW | No. | TW | No. | TW | No. | TW | No. | TW |
| 12 | 1.2471 | 38 | 0.7177 | 17 | 0.3384 | 37 | 0.6795 | 51 | 0.6987 |
| 49 | 0.8806 | 24 | 0.6429 | 41 | 0.1772 | 10 | 0.5365 | 25 | 0.6488 |
| 6 | 1.015 | 8 | 0.5428 | 20 | 0.4256 | 29 | 0.6081 | 46 | 0.612 |
| 27 | 0.9437 | 22 | 0.6414 | 9 | 0.2925 | 42 | 0.635 | 23 | 0.5566 |
| 5 | 0.9235 | 40 | 0.6545 | 50 | 0.3489 | 35 | 0.6686 | 45 | 0.6162 |
| 28 | 0.862 | 30 | 0.5505 | 26 | 0.4283 | 3 | 0.4592 | 18 | 0.5416 |

**The original tumor images of subcutaneous GC mice in five therapy groups.**

Vehicle	5-FU

Combine

Combine+CD8+T depletion	Combine+NK depletion
